# Formation of 1-(thia­zol-2-yl)-4,5-di­hydropyrazoles from simple precursors: synthesis, spectroscopic characterization and the structures of an inter­mediate and two products

**DOI:** 10.1107/S2056989021009312

**Published:** 2021-09-10

**Authors:** Ninganayaka Mahesha, Hemmige S. Yathirajan, Holalagudu A. Nagma Banu, Balakrishna Kalluraya, Ravindranath S. Rathore, Christopher Glidewell

**Affiliations:** a Department of Studies in Chemistry, University of Mysore, Manasagangotri, Mysuru-570 006, India; b Department of Studies in Chemistry, Mangalore University, Mangalagangotri, Mangalore-574199, India; c Department of Bioinformatics, School of Earth, Biological and Environmental Sciences, Central University of South Bihar, Gaya-824236, India; d School of Chemistry, University of St Andrews, St Andrews, Fife KY16 9ST, UK

**Keywords:** heterocyclic compounds, pyrazoles, thia­zoles, synthesis, NMR spectroscopy, crystal structure, mol­ecular structure, hydrogen bonding, supra­molecular assembly

## Abstract

Two new 1-(thia­zol-2-yl)-4,5-di­hydropyrazoles have been synthesized from simple precursors, and characterized both spectroscopically and structurally. In addition, two inter­mediates in the reaction pathway have been isolated and characterized, one of them structurally. The mol­ecules of the inter­mediate (*E*)-1-(4-meth­oxy­phen­yl)-3-[4-(prop-2-yn­yloxy)phen­yl]prop-2-en-1-one, C_19_H_16_O_3_ (I), are linked by a combination of C—H⋯O and C—H⋯π(arene) hydrogen bonds to form ribbons. The products (*RS*)-5-(4-meth­oxy­phen­yl)-1-(4-phenythiazol-2-yl)-3-[4-(prop-2-yn­­yloxy)phen­yl]-4,5-di­hydro-1*H*-pyrazole, C_28_H_23_N_3_O_2_S (II), and (*RS*)-5-(4-meth­oxy­phen­yl)-1-[4-(4-methyl­phen­yl)thia­zol-2-yl]-3-[4-(prop-2-yn­yloxy)phen­yl]-4,5-di­hydro-1*H*-pyrazole, C_29_H_25_N_3_O_2_S (III), are closely related – differing only by presence or absence of a methyl group at the aryl­thia­zolyl substituent – and crystallize in an isomorphous setting. Both mol­ecules contain an effectively planar di­hydro-pyrazole ring, and possess an overall T-shaped structure, which is a characteristic of triaryl-substituted 4,5-di­hydro-1-(thia­zol-2-yl)pyrazole compounds. The crystal packing is characterized by inter­molecular C—H⋯S and C—H⋯π (ar­yl/alkyne) inter­actions. A combination of two C—H⋯π(arene) hydrogen bonds links the product mol­ecules into sheets.

## Abstract 2

(*E*)-1-(4-Meth­oxy­phen­yl)-3-[4-(prop-2-yn­yloxy)phen­yl]prop-2-en-1-one undergoes a cyclo­condensation reaction with thio­semicarbazide to form the corresponding 4,5-di­hydro­pyrazole-1-arbo­thio­amide, which in turn undergoes further cyclo­addition with phenacyl bromides to form 4,5-di­hydro-1-(thia­zol-2-yl)pyrazoles. The mol­ecules adopt an overall T-shape structure. Different combinations of hydrogen bonds link the mol­ecules into ribbons or sheets.

## Chemical context   

Pyrazole derivatives are an important class of *N*-heterocyclic compounds with a wide spectrum of biological activities including anti­bacterial (Song *et al.*, 2013 ▸; Yan *et al.*, 2015 ▸), anti­fungal (Gondru *et al.*, 2015 ▸), anti-inflammatory (El-Sayed *et al.*, 2012 ▸; Kadambar *et al.*, 2021 ▸), anti­microbial (Manju, Kalluraya & Kumar, 2019 ▸) and anti­tumor (Insuasty *et al.*, 2010 ▸; Alam *et al.*, 2016 ▸) activities. Thia­zole derivatives similarly also exhibit a broad spectrum of biological activity, including anti­cancer (Bansal *et al.*, 2020 ▸), anti-inflammatory (Sharma *et al.*, 1998 ▸) and anti­microbial (Kalluraya *et al.*, 2001 ▸) activity.

**Figure 1:**
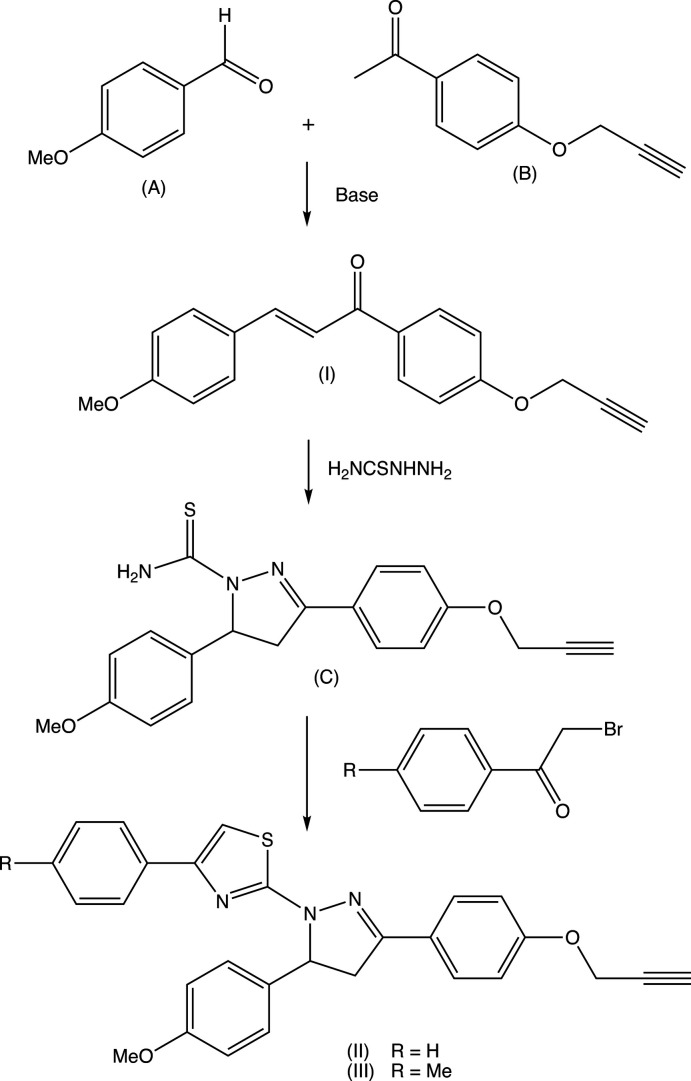
The reaction sequence leading to the formation of compounds (I)–(III).

Accordingly, we have sought to combine pyrazole and thia­zole pharmacophores in a single mol­ecular skeleton and synthesized triaryl-substituted (thia­zol-2-yl)pyrazole compounds (C3,C5-aryl substitutions on the pyrazole ring and C4-aryl substitution on the thia­zole ring). We report here the synthesis of 1-(thia­zolol-2-yl)-4,5-di­hydropyrazoles from simple precursors. The reaction sequence is summarized in Fig. 1 ▸: a base-catalysed condensation reaction between 4-meth­oxy­benzaldehyde (*A*) and a substituted aceto­phenone (*B*) yields the chalcone inter­mediate (I) (Shaibah *et al.*, 2020 ▸). Compound (I) undergoes a cyclo­condensation reaction with a thio­semicarbazide to provide thio­amide inter­mediate (*C*), which in turn undergoes a further cyclo­condensation reaction with a phenacyl bromide to give the thia­zolyl-di­hydro­pyrazoles (II) and (III) (Manju, Kalluraya, Asma *et al.*, 2019 ▸).

Few triaryl-substituted (thia­zol-2-yl)pyrazoles have previously been synthesized and characterized. The synthesis and crystal structure of a new thia­zolyl-pyrazoline derivative bearing the 1,2,4-triazole moiety has been reported (CSD refcode BAKLOQ; Zeng *et al.*, 2012 ▸). A new series of 1,3-thia­zole integrated pyrazoline scaffolds have been synthesized and characterized (DADQIL, DADQEH; Salian *et al.*, 2017 ▸). The synthesis, fluorescence, TGA and crystal structure of a thia­zolyl-pyrazoline derived from chalcones has been described (JUNRAN; Suwunwong *et al.*, 2015 ▸). In addition, the following crystal structures of related compounds have been reported: 2-[3-(4-bromo­phen­yl)-5-(4-fluoro­phen­yl)-4,5-di­hydro-1*H*-pyrazol-1-yl]-4-phenyl-1,3-thia­zole (IDOMOF; Abdel-Wahab *et al.*, 2013*c* ▸), 2-[5-(4-fluoro­phen­yl)-3-(4-meth­yl­phen­yl)-4,5-di­hydro-1*H*-pyrazol-1-yl]-4-phenyl-1,3-thia­zol (MEWQUC; Abdel-Wahab *et al.*, 2013*a* ▸), 2-[3-(4-chloro­phen­yl)-5-(4-fluoro­phen­yl)-4,5-di­hydro-1*H*-pyrazol-1-yl]-4-phenyl-1,3-thia­zole (WIGQIO; Abdel-Wahab *et al.*, 2013*b* ▸), 2-[3-(4-chloro­phen­yl)-5-(4-fluoro­phen­yl)-4,5-di­hydro-1*H*-pyra­zol-1-yl]-8*H*-indeno­[1,2-*d*]thia­zole (WOCFEC; El-Hiti *et al.*, 2019 ▸) and 2-[3-(4-bromo­phen­yl)-5-(4-fluoro­phen­yl)-4,5-di­hydro-1*H*-pyrazol-1-yl]-8*H*-indeno­[1,2-*d*]thia­zole (PUVVAG; Alotaibi *et al.*, 2020 ▸).
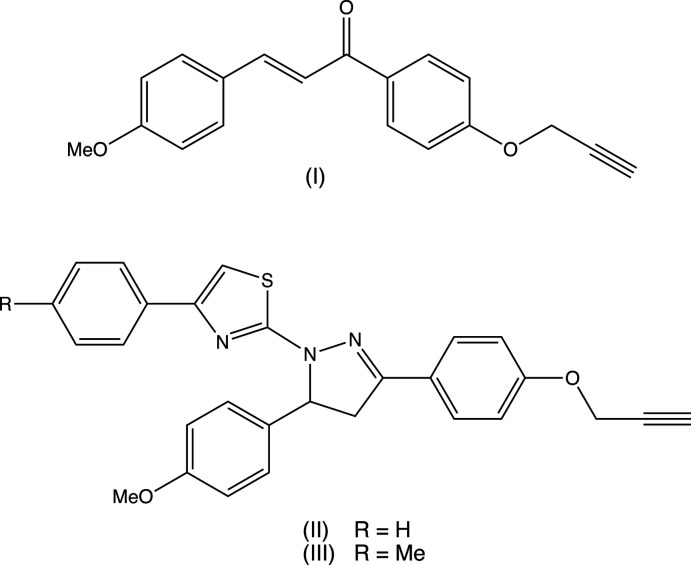


**Figure 2:**
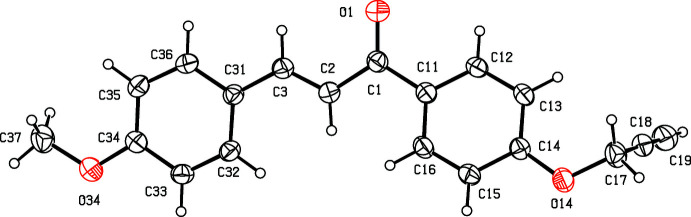
The mol­ecular structure of compound (I) showing the atom-labelling scheme. Displacement ellipsoids are drawn at the 30% probability level.

**Figure 3:**
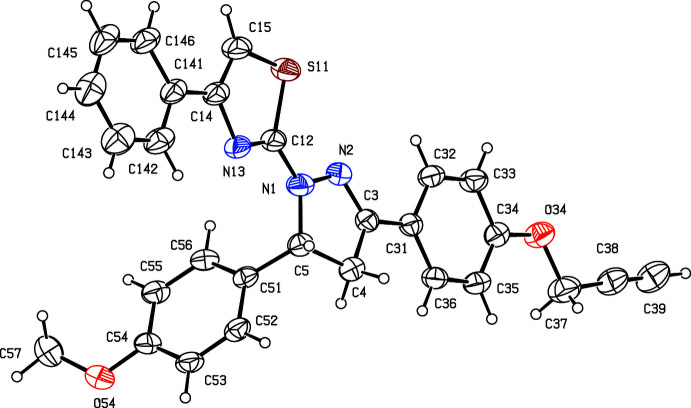
The mol­ecular structure of compound (II) showing the atom-labelling scheme. Displacement ellipsoids are drawn at the 30% probability level.

The proposed synthetic route, as also applied to synthesize many of the aforementioned related compounds, was selected because in some cases, we have introduced mesoionic moieties like sydnone as a part of the triaryl. These sydnones are somewhat sensitive towards vigorous reaction conditions. Under the present conditions selected, the products are stable and the reactions gave reasonably good yields. The chosen synthetic routes of the reported compounds in this study are straightforward with limited steps and readily accessible, cheap starting materials, and yields are reasonably high (Nayak *et al.*, 2013 ▸; Bansal *et al.*, 2020 ▸). The biological activities of few of the related triaryl-substituted (thia­zol-2-yl)pyrazole compounds have been reported in the literature, such as Salian *et al.* (2017 ▸) have demonstrated radical scavenging capacity owing to the destabilization of the radical formed during oxidation. In the present study, compounds (I)–(III) and the inter­mediate (*C*) have been characterized spectroscopically. Chalcone inter­mediate (I) (Fig. 2 ▸) and the di­hydro­(thia­zol­yl)pyrrazole products (II) and (III) (Figs. 3 ▸ and 4 ▸) have also been characterized, and their structures will be described here.

## Structural commentary   

For the thia­zolyl­pyrazole products (II) and (III), and for the inter­mediates (I) and (*C*) (Fig. 1 ▸), the ^1^H NMR spectra contained all of the expected signals (Section 5). In particular, the spectra of each of (I), (II) and (III) contained signals from an ABX spin system arising from the H atoms bonded to atoms C4 and C5 (Figs. 2 ▸ and 3 ▸), consistent with the formation of a new 4,5-di­hydro­pyrazole ring.

In the structure of the chalcone inter­mediate (I) (Fig. 2 ▸), the two aryl rings are both twisted away from the plane of the central spacer unit, atoms C11, C1, O1, C2, C3, C31 [maximum planar deviation of 0.033 (2) Å for C3 atom]. The dihedral angles between this spacer unit and the rings (C11–C16) and (C31–C36) are 21.48 (7) and 8.98 (7)°, respectively, while the dihedral angle between the (C11–C16) ring and the prop-2-yn­yloxy unit (O14, C17, C18, C19) is 73.48 (13)°. The mol­ecule of (I) exhibits no inter­nal symmetry and so is conformationally chiral, but the centrosymmetric space group confirms that equal numbers of the two conformational enanti­omers are present.

Compounds (II) and (III), differing only in the presence or absence of a methyl group at the aryl­thia­zolyl substituent, and are isomorphous and isostructural (Fig. 1 ▸ and Table 2 ▸). In the mol­ecules of (II) and (III), there is a stereogenic centre at atom C5 and, for each, the reference mol­ecule was selected as one having the *R*-configuration at atom C5. However, the space group confirms that both compounds have crystallized as racemic mixtures: this is as expected, as the synthesis of (II) and (III) involves no reagents that could plausibly induce enanti­oselectivity. In each of these compounds, the di­hydro-pyrazole ring is effectively planar (Alex & Kumar, 2014 ▸). The maximum deviations from the mean planes through the ring atoms are 0.44 (3) Å for atom C4 in (II) and only 0.012 (2) Å for atom C3 in (III). The di­hydro-pyrazole ring has been found to be effectively planar among triaryl-substituted (thia­zol-2-yl)pyrazole compounds available in the literature (see *Chemical context* and *Database survey* for references).

In each of (I)–(III), the meth­oxy C atom is coplanar with the adjacent aryl ring [the maximum deviation of atom C37 in (I) and C57 in (II) and (III) from the respective planes are 0.003 (2), 0.529 (5) and 0.405 (7) Å, respectively).

**Figure 5:**
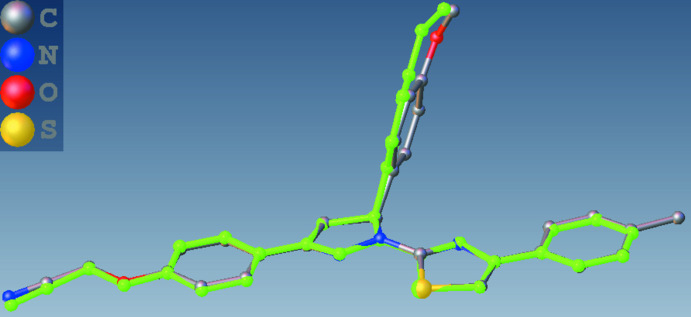
Superimposed image of (II) (shown in green) and (III).

Associated with this coplanarity, the values of the two exocyclic C—C—O angles, at atom C34 in (I) and at atom C54 in each of (II) and (III), differ by *ca* 10°, as typically found in planar alk­oxy­arenes (Seip & Seip, 1973 ▸; Ferguson *et al.*, 1996 ▸; Kiran Kumar, Yathirajan, Foro *et al.*, 2019 ▸; Kiran Kumar *et al.*, 2020 ▸). Overall, both the mol­ecules (II) and (III) adopt a T-shaped structure with the pyrazole C5-substituent anisyl units forming the blade. The remaining part of mol­ecule, the thia­zolyl-pyrazole ring and its substituents form a more or less planar structure, which constitutes the stock of the T-shape. The angle between the plane of the anisyl unit and the remaining part of mol­ecule is 71.8 (1) and 75.3 (1)° in (II) and (III), respectively. Both mol­ecules adopt a more or less similar conformation and a superimposed image of (II) and (III) is shown in Fig. 5 ▸.

## Supra­molecular features   

**Figure 6:**
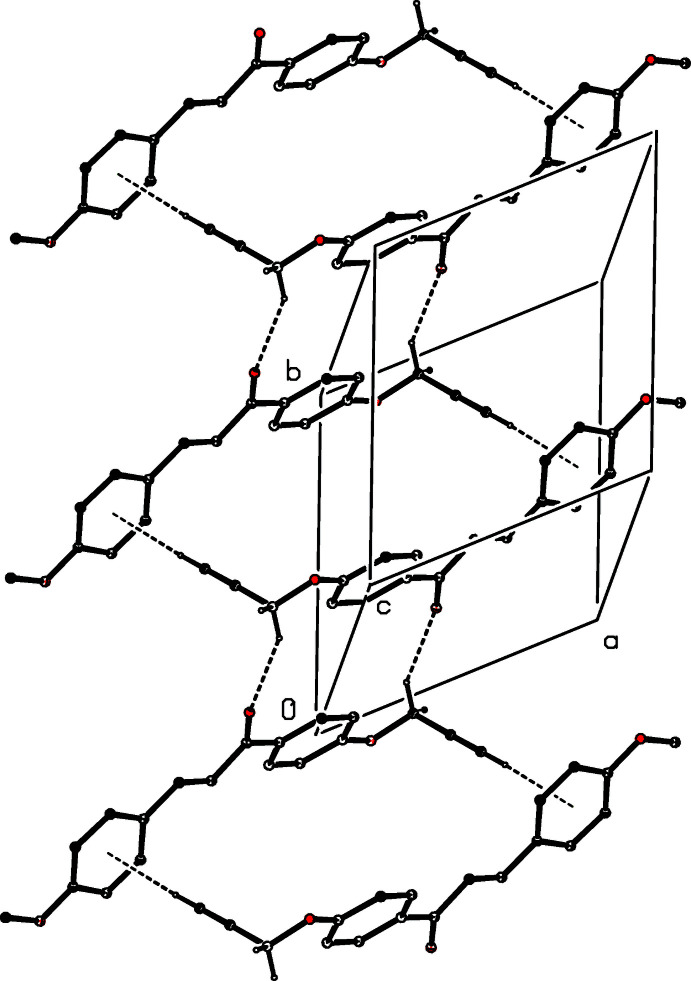
Part of the crystal structure of compound (I) showing the formation of a hydrogen-bonded ribbon of centrosymmetric rings running parallel to the [010] direction. Hydrogen bonds are drawn as dashed lines and, for the sake of clarity, the H atoms bonded to the C atoms which are not involved in the motifs shown have been omitted.

The supra­molecular assembly of the chalcone (I) depends upon two hydrogen-bond-like inter­actions, one each of the C—H⋯O and the C—H⋯π(arene) type (Table 1 ▸). The mol­ecules of (I) are linked into a ribbon of centrosymmetric rings running parallel to the [010] direction (Fig. 6 ▸), in which (propyn­yloxy-CH_2_) C17—H17*B*⋯O1 (carbon­yl) bonded 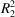
(18) (Etter, 1990 ▸; Etter *et al.*, 1990 ▸; Bernstein *et al.*, 1995 ▸) rings centred at (0, *n*, 0.5) alternate with rings built from (propyn­yloxy-alkyne) C19—H19⋯π (arene of anis­yl) hydrogen bonds, which are centred at (0, *n* + 0.5, 0.5), where *n* represents an integer in each case. The C—H(alkyne)⋯π inter­action has been examined by Holme *et al.* (2013 ▸). Another (propyn­yloxy-phen­yl) C12—H12⋯π (arene of anis­yl) inter­action is also observed.

**Figure 7:**
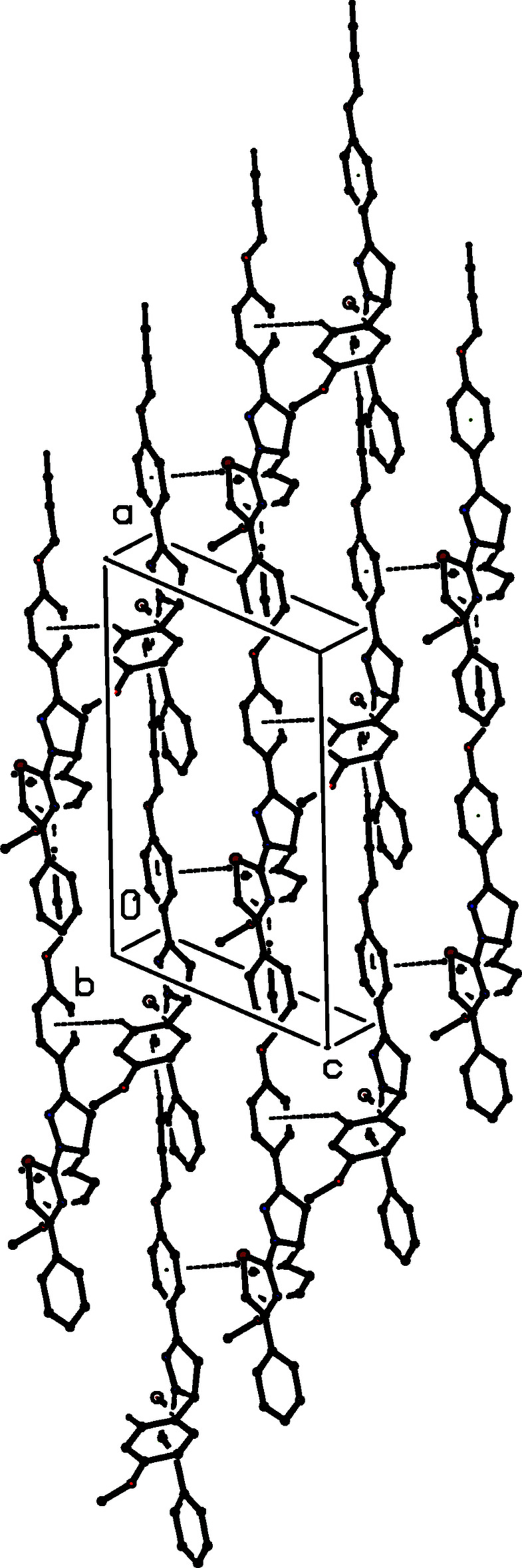
Part of the crystal structure of compound (II) showing the formation of a hydrogen-bonded sheet lying parallel to (010). Hydrogen bonds are drawn as dashed lines and, for the sake of clarity, the H atoms which are not involved in the motifs shown have been omitted.

The structure of compound (II) and (III) contains two C—H⋯π(arene) hydrogen bonds, namely, (propyn­yloxy-alkyne) C39—H39⋯*Cg*2 (arene of anis­yl) and (anisyl-C_ar_H) C56—H56⋯*Cg*1(propyn­yloxy-phen­yl). Together, the two inter­actions generate a sheet (Fig. 7 ▸) lying parallel to (010) in the domain 0 < *y* < 0.5. The inter­action is augmented by a (propyn­yloxy-phen­yl) C35—H35⋯S11 inter­action (Ghosh *et al.*, 2020 ▸) in (III). In (II) too, there is a short H35⋯S11 contact of 2.96 Å; however, it is only 0.04 Å shorter than the sum of van der Waals radii of the corresponding atoms. A second sheet of this type, related to the first by the action of the glide planes lies in the domain 0.5 < *y* < 1.0, but there are no direction-specific inter­actions between adjacent sheets. With the exception of this, there are no significant differences in the packing of (II) and (III).

In (III), a C5—H5⋯π(alkyne) inter­action, also referred as a T-shaped C—H⋯π inter­action (McAdam *et al.*, 2012 ▸) is observed, with the shortest H5⋯C38^i^ [symmetry code: (i) −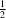 + *x*, 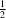 − *y*, 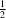 + *z*] distance being 2.74 Å and a C5—H5⋯C38 angle of 159°. In (II), two such short contacts of the C—H⋯π(alkyne) type are observed, with H4*A*⋯C39^i^ and H5⋯C38^i^ distances of 2.80 and 2.81 Å, respectively, which are only 0.10 and 0.09 Å shorter than the sum of of corresponding van der Waals radii.

Additional short intra­molecular C—H⋯O and C—H⋯N contacts are observed in (I)–(III). The packing is devoid of C(alkyne)—H⋯O hydrogen bonding, and no noticeable π–π inter­actions are observed.

## Database survey   

We briefly compare the structures reported here with those of some related compounds. A search for triaryl-substituted (thia­zol-2-yl)pyrazoles in the Cambridge Structural Database (Version 2021.1; Groom *et al.*, 2016 ▸) yielded nine structures that have C3,C5-aryl substitutions in the pyrazole ring and C4-aryl substitution in the thia­zole ring, CSD entries: BAKLOQ, DADQEH, DADQIL, IDOMOF, JUNRAN, MEWQUC, WIGQIO, WOCFEC and PUVVAG (for references, see *Chemical context*). BAKLOQ, and PUVVAG have fused thia­zol and phenyl rings. All these structures are characterized by a T-shaped structure with pyrazole C5-aryl substituents forming its blade and the remaining part of the mol­ecule, the thia­zol-2-yl-pyrazole ring and its substituents, forming a more or less planar structure, which constitutes the stock of the T-shape. Classical hydrogen bonding is not observed in any of these compounds. The di­hydro­pyrazole rings are effectively planar in all these compounds.

Finally, we note that the Cambridge Structural Database (Groom *et al.*, 2016 ▸) records 55 chalcone structures, which were determined as part of the long-time collaboration between the Yathirajan group and the late Professor Jerry P. Jasinski.

## Synthesis and crystallization   

All reagents were obtained commercially, and all were used as received. For the synthesis of compound (I), 4-meth­oxy­benzaldehyde (*A*), (see Fig. 1 ▸) (1.80 g, 0.014 mol) was added to a well-stirred solution of 4-(prop-2-yn­yloxy)aceto­phenone (*B*) (2.00 g, 0.012 mol) and potassium hydroxide (0.90 g, 0.017 mol) in ethanol (10 ml), and this resulting mixture was stirred at ambient temperature for 5 h. When the reaction was complete, as judged from TLC, the mixture was poured into an excess of ice-cold water and the resulting solid product (I) was collected by filtration and crystallized from a mixture of ethanol and *N*,N-di­methyl­formamide (3:2, *v*/*v*) (Shaibah *et al.*, 2020 ▸). Yield 88%, m.p. 375–378 K. IR (cm^−1^) 2180 (alkyne), 1667 (C=O), 1620 (C=C). NMR (CDCl_3_) δ(^1^H) 2.79 (2H, *d*, *J* = 1.8 Hz O-CH_2_), 6.67 (1H, *d*, *J* = 15.6 Hz) (H-2) and 7.54 (1H, *d*, *J* = 15.6 Hz) (H-3), 7.06 (2H, *d*, *J* = 8.8Hz) and 7.16 (2H, *d*, *J* = 8.8Hz) (–C_6_H_4_–), 7.12–7.24 (4H, *m*, –C_6_H_4_–).

For the synthesis of compounds (II) and (III), the precursor chalcone was first converted to the carbo­thio­amide inter­mediate (*C*): thio­semicarbazide (0.155 g, 1.50 mmol) was added to a suspension of (I) (0.50 g, 1.0 mmol) and potassium hydroxide (0.14 g, 2.5 mmol) in ethanol (10 ml). This mixture was then heated under reflux for 8 h, after which time the reaction was judged from TLC to be complete. The mixture was poured onto crushed ice and the resulting solid inter­mediate (*C*) was collected by filtration and crystallized from a mixture of ethanol and *N*,*N*-di­methyl­formamide (3:2, *v*/*v*). Yield 79%, m.p. 422–423 K. Analysis: found C 65.8, H 5.2, N 11.5%; C_20_H_15_N_3_O_2_S requires C 65.7, H 5.2, N 11.5%. IR (cm^−1^) 3339 (NH_2_), 2120 (alkyne). ^1^H NMR (DMSO-*d*_6_) δ 3.09 (1H, *dd*, *J* = 17.5 Hz and 3.2 Hz) and 3.71 (1H, *dd*, *J* = 17.5 Hz and 11.5 Hz) (pyrazole CH_2_), 3.69 (1H, *t*, *J* = 2.3 Hz, alkynic CH), 3.78 (3H, *s*, OMe), 4.52 (2H, *d*, *J* = 2.3 Hz, O-CH_2_), 5.76 (1H, *dd*, *J* = 11.5 Hz and 3.2 Hz, pyrazole CH), 6.75 (2H, *d*, *J* = 8.8 Hz) and 7.02 (2H, *d*, *J* = 8.8 Hz) (–C_6_H_4_–), 7.13 (2H, *d*, *J* = 8,1 Hz) and 7.64 (2H, *d*, *J* = 8.1 Hz) (–C_6_H_4_–). Mixtures of this inter­mediate (1.00 g, 2.0 mmol) and either phenacyl bromide (0.5 g, 2.0 mmol) for (II) or 4-methyl­phenacyl bromide (0.58 g, 2.0 mmol) for (III) in ethanol (20 ml) were heated under reflux for 1 h. The mixtures were then allowed to cool to ambient temperature and the resulting solid products were collected by filtration and then crystallized from mixtures of ethanol and *N*,*N*-di­methyl­formamide (3:2, *v*/*v*) (Manju, Kalluraya, Asma *et al.*, 2019 ▸). Compound (II), yield 88%, m.p. 435–438 K. IR (cm^−1^ 2198 (alkyne), 1618 (C=N), 1600 (C=C). ^1^H NMR (CDCl_3_) δ 2.41 (1H, *t*, *J* = 1.8 Hz), H-39), 3.46 (1H, *dd*, *J* = 16.9 Hz and 5.2 Hz) and 4.10 (1H, *dd*, *J* = 16.9 Hz and 12.4 Hz) (pyrazole CH_2_), 3.90 (3H, *s*, OMe), 4.56 (2H, *d*, *J* = 1.8 Hz, O-CH_2_), 5.43 (1H, *dd*, *J* = 12.4 Hz and 5.2 Hz, pyrazole CH), 6.95 (2H, *d*, *J* = 8.8 Hz) and 7.20 (2H, *d*, *J* = 8.8Hz, –C_6_H_4_–) 7.26–7.63 (9H, *m*, ar­yl), 7.90 (1H, *s*, H-15). Compound (III), yield 82%, m.p. 453–455 K. IR (cm^−1^) 2210 (alkyne), 1620 (C=N), 1605 (C=C). ^1^H NMR (CDCl_3_) δ 2,32 (3H, *s*, C—CH_3_), 2.54 (1H, *t*, *J* = 2.0 Hz), H-39), 3.28 (1H, *dd*, *J* = 17.0 Hz and 6.4 Hz) and 3.84 (1H, *dd*, *J* = 17.0 Hz and 11.8 Hz) (pyrazole CH_2_), 3.77 (3H, *s*, OMe), 4.75 (2H, *d*, *J* = 2.0 Hz, O—CH_2_), 5.69 (1H, *dd*, *J* = 11.8 Hz and 5.4 Hz, pyrazole CH), 6.86 (2H, *d*, *J* = 8.8 Hz), 7.01 (2H, *d*, *J* = 8.8 Hz), 7.11 (2H, *d*, *J* = 8.8 Hz), 7.34 (2H, *d*, *J* = 8.8Hz), 7.57 (2H, *d*, *J* = 8.8 Hz) and 7.72 (2H, *d*, *J* = 8.8 Hz) (3 × –C_6_CH_4_–), 8.00 (1H, *s*, H-15). Crystals of compounds (I)–(III) that were suitable for single-crystal X-ray diffraction were selected directly from the prepared samples.

## Refinement   

Crystal data, data collection and refinement details are summarized in Table 2 ▸. A number of low-angle reflections, which had been attenuated by the beam stop, were omitted from the data sets: for (I), (100), (011), (0
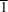
1), (110) and (111); for (II), (11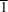), (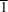
11) and (200); and for (III), (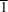
11) and (200). All H atoms were located in difference maps and they were then treated as riding atoms in geometrically idealized positions with C—H distances of 0.98 Å (saturated aliphatic C—H), 0.97 Å (CH_2_), 0.96 Å (CH_3_) or 0.93 Å for all other H atoms, and with *U*_iso_(H) = *kU*_eq_(C), where *k* = 1.5 for the methyl groups, which were permitted to rotate but not to tilt, and *k* = 1.2 for all other H atoms. For compounds (II) and (III), the correct orientation of the structures with respect to the polar axis directions was established by means of the Flack *x* parameter (Flack, 1983 ▸), calculated using quotients of the type (*I* ^+^) - (*I* ^−^)]/[(*I* ^+^) + (*I* ^−^)] (Parsons *et al.*, 2013 ▸). For (II), *x* = 0.00 (3), calculated using 1715 quotients, and for (III) *x* = −0.01 (3), calculated using 1613 quotients.

## Supplementary Material

Crystal structure: contains datablock(s) global, I, II, III. DOI: 10.1107/S2056989021009312/zl5017sup1.cif

Structure factors: contains datablock(s) I. DOI: 10.1107/S2056989021009312/zl5017Isup2.hkl

Structure factors: contains datablock(s) II. DOI: 10.1107/S2056989021009312/zl5017IIsup3.hkl

Structure factors: contains datablock(s) III. DOI: 10.1107/S2056989021009312/zl5017IIIsup4.hkl

Supporting information file. DOI: 10.1107/S2056989021009312/zl5017Isup5.cml

Supporting information file. DOI: 10.1107/S2056989021009312/zl5017IIIsup6.cml

CCDC references: 2108209, 2108208, 2108207

Additional supporting information:  crystallographic information; 3D view; checkCIF report

## Figures and Tables

**Figure 4:**
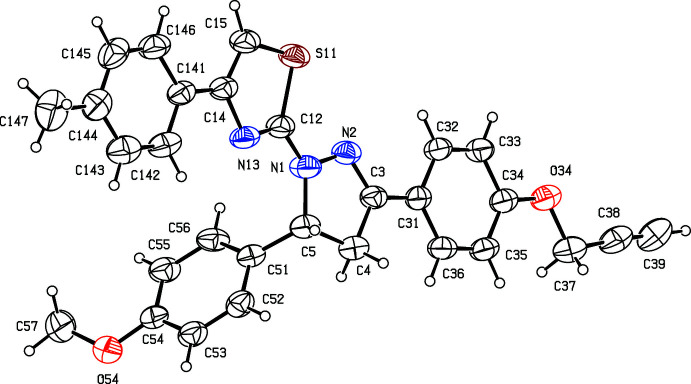
The mol­ecular structure of compound (III) showing the atom-labelling scheme. Displacement ellipsoids are drawn at the 30% probability level.

**Table 1:** Hydrogen-bond parameters (Å, °) *Cg*1 and *Cg*2 represent the centroids of the (C31—C36) and (C51—C56) rings, respectively.

Compound	*D*—H⋯*A*	*D*—H	H⋯*A*	*D*⋯*A*	*D*—H⋯*A*
(I)	C17—H17*B*⋯O1^i^	0.97	2.59	3.456 (2)	148
	C19—H19⋯*Cg*1^ii^	0.93	2.73	3.660 (2)	177
	C12—H12⋯*Cg*1^iii^	0.93	2.89	3.5117 (18)	126
					
(II)	C39—H39⋯*Cg*2^iv^	0.93	2.59	3.365 (5)	141
	C56—H56⋯*Cg*1^v^	0.93	2.91	3.688 (3)	142
					
(III)	C39—H39⋯*Cg*2^iv^	0.93	2.93	3.802 (5)	156
	C56—H56⋯*Cg*1^v^	0.93	2.92	3.689 (3)	141
	C35—H35⋯S11^vi^	0.93	2.86	3.560 (4)	133

Symmetry codes: (i) −*x*, −*y*, 1 − *z*; (ii) −*x*, 1 − *y*, 1 − *z*; (iii) −1 + *x*, *y*, *z*; (iv) 1 + *x*, *y*, *z*; (v) −{1\over 2} + *x*, {1\over 2} − *y*, −{1\over 2} + *z*; (vi) {1\over 2} + *x*, {1\over 2} − *y*, {1\over 2} + *z*.

**Table 2:** Experimental details

	(I)	(II)	(III)
Crystal data			
Chemical formula	C_19_H_16_O_3_	C_28_H_23_N_3_O_2_S	C_29_H_25_N_3_O_2_S
*M*_r_	292.32	465.55	479.58
Crystal system, space group	Triclinic, *P*\overline{1}	Monoclinic, *C* *c*	Monoclinic, *C* *c*
Temperature (K)	297	297	297
*a*, *b*, *c* (Å)	8.6430 (15), 9.9526 (16), 10.0677 (18)	15.7724 (12), 17.6042 (15), 9.3589 (9)	16.5634 (17), 17.7250 (19), 9.4032 (11)
α, β, γ (°)	79.039 (6), 70.124 (6), 68.366 (5)	90, 114.259 (3), 90	90, 116.401 (3), 90
*V* (Å^3^)	755.0 (2)	2369.1 (4)	2472.7 (5)
*Z*	2	4	4
Radiation type	Mo *K*α	Mo *K*α	Mo *K*α
μ (mm^−1^)	0.09	0.17	0.16
Crystal size (mm)	0.16 × 0.15 × 0.12	0.20 × 0.18 × 0.15	0.18 × 0.16 × 0.15

Data collection			
Diffractometer	Bruker D8 Venture	Bruker D8 Venture	Bruker D8 Venture
Absorption correction	Multi-scan (*SADABS*; Bruker, 2016 ▸)	Multi-scan (*SADABS*; Bruker, 2016 ▸)	Multi-scan (*SADABS*; Bruker, 2016 ▸)
*T*_min_, *T*_max_	0.966, 0.969	0.949, 0.975	0.949, 0.976
No. of measured, independent and observed [*I* > 2σ(*I*)] reflections	45325, 5029, 3072	46650, 6087, 4331	40416, 5578, 3802
*R*_int_	0.066	0.062	0.058
(sin θ/λ)_max_ (Å^−1^)	0.735	0.692	0.652

Refinement			
*R*[*F*^2^ > 2σ(*F* ^2^)], *wR*(*F* ^2^), *S*	0.053, 0.162, 1.01	0.040, 0.103, 1.05	0.042, 0.121, 1.08
No. of reflections	5029	6087	5578
No. of parameters	200	308	318
No. of restraints	0	2	2
H-atom treatment	H-atom parameters constrained	H-atom parameters constrained	H-atom parameters constrained
Δρ_max_, Δρ_min_ (e Å^−3^)	0.36, −0.20	0.12, −0.16	0.15, −0.17
Absolute structure	–	Flack *x* determined using 1715 quotients [(*I* ^+^)−(*I* ^−^)]/[(*I* ^+^)+(*I* ^−^)] (Parsons *et al.*, 2013 ▸)	Flack *x* determined using 1613 quotients [(*I* ^+^)−(*I* ^−^)]/[(*I* ^+^)+(*I* ^−^)] (Parsons *et al.*, 2013 ▸)
Absolute structure parameter	–	0.00 (3)	−0.01 (3)

Computer programs: *APEX3* (Bruker, 2016 ▸), *APEX3*, *SAINT* and *XPREP* (Bruker, 2016 ▸), *SHELXT2014/5* (Sheldrick, 2015*a* ▸), *SHELXL2014* (Sheldrick, 2015*b* ▸) and *PLATON* (Spek, 2020 ▸).

## supplementary crystallographic information

### (E)-1-(4-Methoxyphenyl)-3-[4-(prop-2-ynyloxy)phenyl]prop-2-en-1-one (I) Crystal data

**Table:**

C_19_H_16_O_3_	*Z* = 2
*M**_r_* = 292.32	*F*(000) = 308
Triclinic, *P*1	*D*_x_ = 1.286 Mg m^−^^3^
*a* = 8.6430 (15) Å	Mo *K*α radiation, λ = 0.71073 Å
*b* = 9.9526 (16) Å	Cell parameters from 5248 reflections
*c* = 10.0677 (18) Å	θ = 2.7–32.5°
α = 79.039 (6)°	µ = 0.09 mm^−^^1^
β = 70.124 (6)°	*T* = 297 K
γ = 68.366 (5)°	Block, colourless
*V* = 755.0 (2) Å^3^	0.16 × 0.15 × 0.12 mm

### (E)-1-(4-Methoxyphenyl)-3-[4-(prop-2-ynyloxy)phenyl]prop-2-en-1-one (I) Data collection

**Table:**

Bruker D8 Venture diffractometer	5029 independent reflections
Radiation source: INCOATEC high brilliance microfocus sealed tube	3072 reflections with *I* > 2σ(*I*)
Multilayer mirror monochromator	*R*_int_ = 0.066
φ and ω scans	θ_max_ = 31.5°, θ_min_ = 2.9°
Absorption correction: multi-scan (SADABS; Bruker, 2016)	*h* = −12→12
*T*_min_ = 0.966, *T*_max_ = 0.969	*k* = −14→14
45325 measured reflections	*l* = −14→14

### (E)-1-(4-Methoxyphenyl)-3-[4-(prop-2-ynyloxy)phenyl]prop-2-en-1-one (I) Refinement

**Table:**

Refinement on *F*^2^	Primary atom site location: difference Fourier map
Least-squares matrix: full	Hydrogen site location: inferred from neighbouring sites
*R*[*F*^2^ > 2σ(*F*^2^)] = 0.053	H-atom parameters constrained
*wR*(*F*^2^) = 0.162	*w* = 1/[σ^2^(*F*_o_^2^) + (0.0578*P*)^2^ + 0.2039*P*] where *P* = (*F*_o_^2^ + 2*F*_c_^2^)/3
*S* = 1.01	(Δ/σ)_max_ < 0.001
5029 reflections	Δρ_max_ = 0.36 e Å^−^^3^
200 parameters	Δρ_min_ = −0.20 e Å^−^^3^
0 restraints	

### (E)-1-(4-Methoxyphenyl)-3-[4-(prop-2-ynyloxy)phenyl]prop-2-en-1-one (I) Special details

**Table:**

Geometry. All esds (except the esd in the dihedral angle between two l.s. planes) are estimated using the full covariance matrix. The cell esds are taken into account individually in the estimation of esds in distances, angles and torsion angles; correlations between esds in cell parameters are only used when they are defined by crystal symmetry. An approximate (isotropic) treatment of cell esds is used for estimating esds involving l.s. planes.

### (E)-1-(4-Methoxyphenyl)-3-[4-(prop-2-ynyloxy)phenyl]prop-2-en-1-one (I) Fractional atomic coordinates and isotropic or equivalent isotropic displacement parameters (Å^2^)

**Table:**

	*x*	*y*	*z*	*U*_iso_*/*U*_eq_	
C1	0.35447 (18)	0.15799 (16)	0.42144 (16)	0.0472 (3)	
O1	0.36622 (16)	0.11332 (14)	0.31105 (12)	0.0652 (3)	
C2	0.47763 (19)	0.22583 (17)	0.42604 (16)	0.0498 (3)	
H2	0.4598	0.2643	0.5094	0.060*	
C3	0.61295 (19)	0.23433 (16)	0.31606 (16)	0.0480 (3)	
H3	0.6285	0.1928	0.2351	0.058*	
C11	0.21298 (18)	0.14699 (14)	0.55306 (15)	0.0436 (3)	
C12	0.06427 (19)	0.13056 (16)	0.54368 (16)	0.0469 (3)	
H12	0.0557	0.1266	0.4550	0.056*	
C13	−0.07138 (19)	0.11992 (16)	0.66299 (16)	0.0485 (3)	
H13	−0.1707	0.1106	0.6545	0.058*	
C14	−0.05751 (18)	0.12334 (15)	0.79527 (15)	0.0456 (3)	
C15	0.0910 (2)	0.13783 (17)	0.80677 (16)	0.0514 (3)	
H15	0.1007	0.1390	0.8957	0.062*	
C16	0.22343 (19)	0.15049 (16)	0.68757 (16)	0.0496 (3)	
H16	0.3216	0.1616	0.6965	0.060*	
O14	−0.18261 (15)	0.11348 (14)	0.92107 (11)	0.0599 (3)	
C17	−0.3326 (2)	0.0850 (2)	0.91866 (19)	0.0601 (4)	
H17A	−0.3898	0.0520	1.0133	0.072*	
H17B	−0.2950	0.0076	0.8573	0.072*	
C18	−0.4578 (2)	0.2122 (2)	0.86912 (17)	0.0562 (4)	
C19	−0.5601 (3)	0.3130 (2)	0.8308 (2)	0.0709 (5)	
H19	−0.6414	0.3932	0.8004	0.085*	
C31	0.74006 (18)	0.30161 (15)	0.30821 (14)	0.0439 (3)	
C32	0.72813 (19)	0.37748 (16)	0.41724 (15)	0.0485 (3)	
H32	0.6349	0.3862	0.4995	0.058*	
C33	0.8511 (2)	0.43923 (17)	0.40517 (16)	0.0513 (3)	
H33	0.8398	0.4896	0.4787	0.062*	
C34	0.9922 (2)	0.42693 (16)	0.28368 (16)	0.0485 (3)	
C35	1.0071 (2)	0.35335 (18)	0.17388 (16)	0.0551 (4)	
H35	1.1006	0.3448	0.0918	0.066*	
C36	0.8814 (2)	0.29253 (18)	0.18739 (16)	0.0535 (4)	
H36	0.8921	0.2438	0.1129	0.064*	
O34	1.10894 (16)	0.48771 (14)	0.28515 (13)	0.0648 (3)	
C37	1.2544 (3)	0.4817 (2)	0.1633 (2)	0.0728 (5)	
H37A	1.3212	0.3823	0.1449	0.109*	
H37B	1.3259	0.5277	0.1792	0.109*	
H37C	1.2145	0.5310	0.0833	0.109*	

### (E)-1-(4-Methoxyphenyl)-3-[4-(prop-2-ynyloxy)phenyl]prop-2-en-1-one (I) Atomic displacement parameters (Å^2^)

**Table:**

	*U*^11^	*U*^22^	*U*^33^	*U*^12^	*U*^13^	*U*^23^
C1	0.0429 (7)	0.0456 (7)	0.0533 (8)	−0.0111 (6)	−0.0155 (6)	−0.0091 (6)
O1	0.0602 (7)	0.0829 (8)	0.0585 (7)	−0.0276 (6)	−0.0111 (5)	−0.0229 (6)
C2	0.0463 (8)	0.0541 (8)	0.0498 (8)	−0.0162 (6)	−0.0111 (6)	−0.0119 (6)
C3	0.0460 (7)	0.0493 (8)	0.0485 (8)	−0.0130 (6)	−0.0136 (6)	−0.0097 (6)
C11	0.0419 (7)	0.0390 (6)	0.0507 (8)	−0.0105 (5)	−0.0160 (6)	−0.0067 (5)
C12	0.0489 (8)	0.0475 (7)	0.0503 (8)	−0.0157 (6)	−0.0201 (6)	−0.0078 (6)
C13	0.0458 (7)	0.0528 (8)	0.0556 (8)	−0.0203 (6)	−0.0204 (6)	−0.0055 (6)
C14	0.0444 (7)	0.0463 (7)	0.0482 (7)	−0.0164 (6)	−0.0167 (6)	0.0004 (6)
C15	0.0502 (8)	0.0626 (9)	0.0472 (8)	−0.0186 (7)	−0.0228 (6)	−0.0017 (6)
C16	0.0435 (7)	0.0559 (8)	0.0561 (8)	−0.0172 (6)	−0.0212 (6)	−0.0055 (6)
O14	0.0540 (6)	0.0838 (8)	0.0505 (6)	−0.0341 (6)	−0.0190 (5)	0.0056 (5)
C17	0.0565 (9)	0.0690 (10)	0.0619 (10)	−0.0341 (8)	−0.0172 (7)	0.0058 (8)
C18	0.0520 (9)	0.0707 (10)	0.0522 (8)	−0.0287 (8)	−0.0116 (7)	−0.0086 (7)
C19	0.0636 (11)	0.0788 (12)	0.0701 (12)	−0.0172 (9)	−0.0236 (9)	−0.0106 (9)
C31	0.0413 (7)	0.0449 (7)	0.0423 (7)	−0.0104 (5)	−0.0111 (5)	−0.0062 (5)
C32	0.0422 (7)	0.0549 (8)	0.0428 (7)	−0.0098 (6)	−0.0088 (6)	−0.0106 (6)
C33	0.0527 (8)	0.0546 (8)	0.0482 (8)	−0.0136 (7)	−0.0162 (6)	−0.0136 (6)
C34	0.0492 (8)	0.0474 (7)	0.0520 (8)	−0.0160 (6)	−0.0183 (6)	−0.0043 (6)
C35	0.0518 (8)	0.0662 (10)	0.0451 (8)	−0.0241 (7)	−0.0032 (6)	−0.0101 (7)
C36	0.0550 (9)	0.0632 (9)	0.0442 (8)	−0.0224 (7)	−0.0078 (6)	−0.0152 (6)
O34	0.0631 (7)	0.0746 (8)	0.0666 (7)	−0.0335 (6)	−0.0154 (6)	−0.0131 (6)
C37	0.0687 (12)	0.0856 (13)	0.0734 (12)	−0.0436 (10)	−0.0164 (9)	0.0015 (10)

### (E)-1-(4-Methoxyphenyl)-3-[4-(prop-2-ynyloxy)phenyl]prop-2-en-1-one (I) Geometric parameters (Å, º)

**Table:**

C1—O1	1.2332 (18)	C17—H17A	0.9700
C1—C2	1.472 (2)	C17—H17B	0.9700
C1—C11	1.485 (2)	C18—C19	1.170 (3)
C2—C3	1.326 (2)	C19—H19	0.9300
C2—H2	0.9300	C31—C36	1.390 (2)
C3—C31	1.458 (2)	C31—C32	1.402 (2)
C3—H3	0.9300	C32—C33	1.374 (2)
C11—C12	1.3896 (19)	C32—H32	0.9300
C11—C16	1.395 (2)	C33—C34	1.390 (2)
C12—C13	1.384 (2)	C33—H33	0.9300
C12—H12	0.9300	C34—O34	1.3594 (18)
C13—C14	1.385 (2)	C34—C35	1.384 (2)
C13—H13	0.9300	C35—C36	1.385 (2)
C14—O14	1.3721 (17)	C35—H35	0.9300
C14—C15	1.387 (2)	C36—H36	0.9300
C15—C16	1.372 (2)	O34—C37	1.419 (2)
C15—H15	0.9300	C37—H37A	0.9600
C16—H16	0.9300	C37—H37B	0.9600
O14—C17	1.4341 (19)	C37—H37C	0.9600
C17—C18	1.462 (2)		
			
O1—C1—C2	121.19 (14)	O14—C17—H17B	109.0
O1—C1—C11	120.62 (13)	C18—C17—H17B	109.0
C2—C1—C11	118.18 (13)	H17A—C17—H17B	107.8
C3—C2—C1	122.60 (14)	C19—C18—C17	178.89 (19)
C3—C2—H2	118.7	C18—C19—H19	180.0
C1—C2—H2	118.7	C36—C31—C32	116.87 (13)
C2—C3—C31	127.06 (14)	C36—C31—C3	119.67 (13)
C2—C3—H3	116.5	C32—C31—C3	123.46 (13)
C31—C3—H3	116.5	C33—C32—C31	121.46 (13)
C12—C11—C16	117.96 (13)	C33—C32—H32	119.3
C12—C11—C1	119.34 (13)	C31—C32—H32	119.3
C16—C11—C1	122.70 (13)	C32—C33—C34	120.38 (13)
C13—C12—C11	121.65 (13)	C32—C33—H33	119.8
C13—C12—H12	119.2	C34—C33—H33	119.8
C11—C12—H12	119.2	O34—C34—C35	125.05 (14)
C12—C13—C14	119.22 (13)	O34—C34—C33	115.41 (13)
C12—C13—H13	120.4	C35—C34—C33	119.53 (14)
C14—C13—H13	120.4	C34—C35—C36	119.36 (14)
O14—C14—C13	124.69 (13)	C34—C35—H35	120.3
O14—C14—C15	115.39 (13)	C36—C35—H35	120.3
C13—C14—C15	119.92 (14)	C35—C36—C31	122.40 (14)
C16—C15—C14	120.30 (14)	C35—C36—H36	118.8
C16—C15—H15	119.8	C31—C36—H36	118.8
C14—C15—H15	119.8	C34—O34—C37	118.37 (13)
C15—C16—C11	120.93 (13)	O34—C37—H37A	109.5
C15—C16—H16	119.5	O34—C37—H37B	109.5
C11—C16—H16	119.5	H37A—C37—H37B	109.5
C14—O14—C17	118.59 (12)	O34—C37—H37C	109.5
O14—C17—C18	112.83 (14)	H37A—C37—H37C	109.5
O14—C17—H17A	109.0	H37B—C37—H37C	109.5
C18—C17—H17A	109.0		
			
O1—C1—C2—C3	−4.4 (2)	C13—C14—O14—C17	5.7 (2)
C11—C1—C2—C3	176.67 (14)	C15—C14—O14—C17	−174.36 (14)
C1—C2—C3—C31	178.55 (14)	C14—O14—C17—C18	−76.01 (19)
O1—C1—C11—C12	−20.5 (2)	C2—C3—C31—C36	175.71 (15)
C2—C1—C11—C12	158.44 (13)	C2—C3—C31—C32	−4.6 (2)
O1—C1—C11—C16	158.65 (15)	C36—C31—C32—C33	−0.3 (2)
C2—C1—C11—C16	−22.4 (2)	C3—C31—C32—C33	−179.98 (14)
C16—C11—C12—C13	0.9 (2)	C31—C32—C33—C34	−0.4 (2)
C1—C11—C12—C13	−179.94 (13)	C32—C33—C34—O34	−177.78 (13)
C11—C12—C13—C14	−1.1 (2)	C32—C33—C34—C35	0.8 (2)
C12—C13—C14—O14	−179.87 (13)	O34—C34—C35—C36	178.04 (15)
C12—C13—C14—C15	0.2 (2)	C33—C34—C35—C36	−0.3 (2)
O14—C14—C15—C16	−179.15 (13)	C34—C35—C36—C31	−0.4 (3)
C13—C14—C15—C16	0.8 (2)	C32—C31—C36—C35	0.7 (2)
C14—C15—C16—C11	−0.9 (2)	C3—C31—C36—C35	−179.57 (14)
C12—C11—C16—C15	0.1 (2)	C35—C34—O34—C37	2.8 (2)
C1—C11—C16—C15	−179.02 (14)	C33—C34—O34—C37	−178.74 (14)

### (E)-1-(4-Methoxyphenyl)-3-[4-(prop-2-ynyloxy)phenyl]prop-2-en-1-one (I) Hydrogen-bond geometry (Å, º)

**Table:**

*D*—H···*A*	*D*—H	H···*A*	*D*···*A*	*D*—H···*A*
C17—H17*B*···O1^i^	0.97	2.59	3.456 (2)	148
C3—H3···O1	0.93	2.50	2.827 (2)	101
C12—H12···*Cg*1^ii^	0.93	2.89	3.5117 (18)	126
C19—H19···*Cg*1^iii^	0.93	2.73	3.660 (2)	177

Symmetry codes: (i) −*x*, −*y*, −*z*+1; (ii) *x*−1, *y*, *z*; (iii) −*x*, −*y*+1, −*z*+1.

### (RS)-5-(4-Methoxyphenyl)-1-(4-phenythiazol-2-yl)-3-(4-(prop-2-ynyloxy)phenyl)-4,5-dihydro-1H-pyrazole (II) Crystal data

**Table:**

C_28_H_23_N_3_O_2_S	*F*(000) = 976
*M**_r_* = 465.55	*D*_x_ = 1.305 Mg m^−^^3^
Monoclinic, *C**c*	Mo *K*α radiation, λ = 0.71073 Å
*a* = 15.7724 (12) Å	Cell parameters from 6124 reflections
*b* = 17.6042 (15) Å	θ = 2.5–29.7°
*c* = 9.3589 (9) Å	µ = 0.17 mm^−^^1^
β = 114.259 (3)°	*T* = 297 K
*V* = 2369.1 (4) Å^3^	Block, colourless
*Z* = 4	0.20 × 0.18 × 0.15 mm

### (RS)-5-(4-Methoxyphenyl)-1-(4-phenythiazol-2-yl)-3-(4-(prop-2-ynyloxy)phenyl)-4,5-dihydro-1H-pyrazole (II) Data collection

**Table:**

Bruker D8 Venture diffractometer	6087 independent reflections
Radiation source: INCOATEC high brilliance microfocus sealed tube	4331 reflections with *I* > 2σ(*I*)
Multilayer mirror monochromator	*R*_int_ = 0.062
φ and ω scans	θ_max_ = 29.5°, θ_min_ = 3.4°
Absorption correction: multi-scan (SADABS; Bruker, 2016)	*h* = −21→17
*T*_min_ = 0.949, *T*_max_ = 0.975	*k* = −24→24
46650 measured reflections	*l* = −12→12

### (RS)-5-(4-Methoxyphenyl)-1-(4-phenythiazol-2-yl)-3-(4-(prop-2-ynyloxy)phenyl)-4,5-dihydro-1H-pyrazole (II) Refinement

**Table:**

Refinement on *F*^2^	Hydrogen site location: inferred from neighbouring sites
Least-squares matrix: full	H-atom parameters constrained
*R*[*F*^2^ > 2σ(*F*^2^)] = 0.040	*w* = 1/[σ^2^(*F*_o_^2^) + (0.0465*P*)^2^ + 0.3024*P*] where *P* = (*F*_o_^2^ + 2*F*_c_^2^)/3
*wR*(*F*^2^) = 0.103	(Δ/σ)_max_ < 0.001
*S* = 1.05	Δρ_max_ = 0.12 e Å^−^^3^
6087 reflections	Δρ_min_ = −0.16 e Å^−^^3^
308 parameters	Absolute structure: Flack *x* determined using 1715 quotients [(*I*^+^)-(*I*^-^)]/[(*I*^+^)+(*I*^-^)] (Parsons *et al.*, 2013)
2 restraints	Absolute structure parameter: 0.00 (3)
Primary atom site location: difference Fourier map	

### (RS)-5-(4-Methoxyphenyl)-1-(4-phenythiazol-2-yl)-3-(4-(prop-2-ynyloxy)phenyl)-4,5-dihydro-1H-pyrazole (II) Special details

**Table:**

Geometry. All esds (except the esd in the dihedral angle between two l.s. planes) are estimated using the full covariance matrix. The cell esds are taken into account individually in the estimation of esds in distances, angles and torsion angles; correlations between esds in cell parameters are only used when they are defined by crystal symmetry. An approximate (isotropic) treatment of cell esds is used for estimating esds involving l.s. planes.

### (RS)-5-(4-Methoxyphenyl)-1-(4-phenythiazol-2-yl)-3-(4-(prop-2-ynyloxy)phenyl)-4,5-dihydro-1H-pyrazole (II) Fractional atomic coordinates and isotropic or equivalent isotropic displacement parameters (Å^2^)

**Table:**

	*x*	*y*	*z*	*U*_iso_*/*U*_eq_	
N1	0.37305 (18)	0.29886 (13)	0.5843 (3)	0.0698 (7)	
N2	0.44590 (16)	0.30871 (13)	0.5400 (3)	0.0615 (6)	
C3	0.50583 (18)	0.25567 (14)	0.6044 (3)	0.0537 (6)	
C4	0.48110 (19)	0.20499 (17)	0.7111 (4)	0.0657 (7)	
H4A	0.5241	0.2118	0.8197	0.079*	
H4B	0.4809	0.1520	0.6828	0.079*	
C5	0.38250 (19)	0.23200 (15)	0.6834 (3)	0.0597 (6)	
H5	0.3811	0.2475	0.7830	0.072*	
S11	0.31763 (7)	0.43908 (4)	0.46457 (10)	0.0756 (2)	
C12	0.3097 (2)	0.35556 (14)	0.5592 (3)	0.0619 (7)	
N13	0.24061 (17)	0.35145 (12)	0.6003 (3)	0.0640 (6)	
C14	0.1866 (2)	0.41709 (15)	0.5544 (3)	0.0639 (8)	
C15	0.2173 (3)	0.46935 (17)	0.4794 (4)	0.0760 (9)	
H15	0.1880	0.5155	0.4411	0.091*	
C141	0.1032 (2)	0.42337 (16)	0.5869 (4)	0.0668 (8)	
C142	0.0831 (2)	0.36891 (19)	0.6757 (4)	0.0764 (9)	
H142	0.1241	0.3287	0.7180	0.092*	
C143	0.0035 (3)	0.3734 (2)	0.7022 (5)	0.0927 (11)	
H143	−0.0088	0.3358	0.7610	0.111*	
C144	−0.0578 (3)	0.4323 (3)	0.6432 (4)	0.0912 (11)	
H144	−0.1114	0.4348	0.6614	0.109*	
C145	−0.0390 (3)	0.4872 (2)	0.5573 (5)	0.0928 (13)	
H145	−0.0799	0.5277	0.5179	0.111*	
C146	0.0401 (3)	0.48317 (19)	0.5284 (4)	0.0812 (10)	
H146	0.0514	0.5209	0.4689	0.097*	
C31	0.59091 (18)	0.24801 (14)	0.5805 (3)	0.0515 (6)	
C32	0.6202 (2)	0.30393 (16)	0.5045 (3)	0.0601 (7)	
H32	0.5834	0.3467	0.4649	0.072*	
C33	0.7017 (2)	0.29701 (17)	0.4871 (3)	0.0646 (7)	
H33	0.7203	0.3351	0.4375	0.078*	
C34	0.75702 (19)	0.23232 (16)	0.5444 (3)	0.0576 (6)	
C35	0.7292 (2)	0.17629 (15)	0.6179 (3)	0.0614 (7)	
H35	0.7654	0.1329	0.6546	0.074*	
C36	0.6477 (2)	0.18442 (14)	0.6373 (3)	0.0583 (7)	
H36	0.6302	0.1467	0.6894	0.070*	
O34	0.83796 (15)	0.23069 (13)	0.5230 (2)	0.0729 (6)	
C37	0.8963 (3)	0.1658 (2)	0.5840 (5)	0.0892 (11)	
H37A	0.8650	0.1206	0.5278	0.107*	
H37B	0.9090	0.1591	0.6938	0.107*	
C38	0.9823 (3)	0.1766 (2)	0.5668 (4)	0.0791 (9)	
C39	1.0545 (3)	0.1841 (2)	0.5627 (5)	0.0937 (11)	
H39	1.1122	0.1902	0.5595	0.112*	
C51	0.31025 (17)	0.17114 (13)	0.6070 (3)	0.0497 (5)	
C52	0.30167 (19)	0.11341 (16)	0.7020 (3)	0.0593 (6)	
H52	0.3387	0.1145	0.8091	0.071*	
C53	0.2399 (2)	0.05516 (16)	0.6411 (4)	0.0651 (7)	
H53	0.2361	0.0167	0.7063	0.078*	
C54	0.1830 (2)	0.05331 (15)	0.4823 (4)	0.0626 (7)	
C55	0.1922 (2)	0.10879 (15)	0.3862 (3)	0.0621 (7)	
H155	0.1554	0.1073	0.2790	0.075*	
C56	0.2559 (2)	0.16685 (16)	0.4487 (3)	0.0576 (6)	
H56	0.2622	0.2037	0.3824	0.069*	
O54	0.11946 (19)	−0.00466 (13)	0.4328 (3)	0.0955 (8)	
C57	0.0407 (3)	0.0056 (2)	0.2901 (5)	0.1053 (13)	
H57A	0.0590	0.0025	0.2043	0.158*	
H57B	0.0138	0.0545	0.2899	0.158*	
H57C	−0.0042	−0.0333	0.2793	0.158*	

### (RS)-5-(4-Methoxyphenyl)-1-(4-phenythiazol-2-yl)-3-(4-(prop-2-ynyloxy)phenyl)-4,5-dihydro-1H-pyrazole (II) Atomic displacement parameters (Å^2^)

**Table:**

	*U*^11^	*U*^22^	*U*^33^	*U*^12^	*U*^13^	*U*^23^
N1	0.0646 (14)	0.0476 (12)	0.0985 (18)	0.0086 (11)	0.0350 (13)	0.0120 (12)
N2	0.0616 (14)	0.0458 (12)	0.0734 (15)	0.0001 (10)	0.0240 (11)	0.0015 (10)
C3	0.0566 (15)	0.0444 (12)	0.0528 (14)	−0.0031 (11)	0.0152 (11)	−0.0044 (11)
C4	0.0589 (16)	0.0601 (16)	0.0731 (18)	0.0057 (13)	0.0221 (14)	0.0128 (14)
C5	0.0607 (16)	0.0516 (14)	0.0662 (17)	0.0071 (12)	0.0255 (13)	0.0048 (12)
S11	0.0889 (5)	0.0434 (3)	0.0841 (5)	−0.0019 (4)	0.0250 (4)	0.0007 (4)
C12	0.0657 (17)	0.0396 (12)	0.0672 (18)	0.0029 (12)	0.0140 (14)	−0.0030 (12)
N13	0.0640 (15)	0.0442 (12)	0.0744 (16)	0.0111 (10)	0.0189 (12)	0.0000 (10)
C14	0.0722 (18)	0.0423 (13)	0.0574 (16)	0.0104 (12)	0.0066 (13)	−0.0089 (11)
C15	0.089 (2)	0.0418 (14)	0.082 (2)	0.0108 (14)	0.0193 (17)	−0.0013 (14)
C141	0.0698 (18)	0.0490 (15)	0.0601 (16)	0.0152 (13)	0.0050 (13)	−0.0135 (12)
C142	0.085 (2)	0.0637 (18)	0.0719 (19)	0.0242 (15)	0.0232 (17)	−0.0024 (15)
C143	0.101 (3)	0.091 (3)	0.084 (2)	0.020 (2)	0.036 (2)	−0.008 (2)
C144	0.086 (2)	0.096 (3)	0.081 (2)	0.022 (2)	0.0222 (19)	−0.024 (2)
C145	0.081 (2)	0.076 (2)	0.091 (3)	0.0353 (19)	0.0053 (19)	−0.021 (2)
C146	0.083 (2)	0.0587 (17)	0.078 (2)	0.0239 (16)	0.0081 (17)	−0.0047 (15)
C31	0.0580 (15)	0.0446 (12)	0.0453 (13)	−0.0020 (10)	0.0145 (10)	−0.0046 (10)
C32	0.0661 (17)	0.0496 (14)	0.0605 (16)	0.0088 (12)	0.0219 (13)	0.0107 (12)
C33	0.0712 (19)	0.0587 (16)	0.0682 (18)	0.0069 (13)	0.0330 (15)	0.0166 (13)
C34	0.0592 (16)	0.0570 (15)	0.0546 (15)	0.0053 (12)	0.0213 (12)	0.0001 (12)
C35	0.0700 (18)	0.0491 (14)	0.0617 (16)	0.0112 (12)	0.0236 (13)	0.0067 (12)
C36	0.0669 (17)	0.0441 (13)	0.0612 (16)	0.0011 (12)	0.0235 (13)	0.0039 (11)
O34	0.0743 (14)	0.0711 (13)	0.0813 (14)	0.0183 (11)	0.0401 (11)	0.0170 (10)
C37	0.086 (2)	0.076 (2)	0.114 (3)	0.0243 (18)	0.049 (2)	0.025 (2)
C38	0.079 (2)	0.075 (2)	0.087 (2)	0.0260 (17)	0.0390 (19)	0.0087 (16)
C39	0.086 (3)	0.098 (3)	0.105 (3)	0.037 (2)	0.047 (2)	0.008 (2)
C51	0.0518 (13)	0.0479 (12)	0.0539 (13)	0.0127 (10)	0.0264 (11)	0.0081 (10)
C52	0.0574 (15)	0.0658 (16)	0.0539 (14)	0.0118 (13)	0.0221 (12)	0.0191 (12)
C53	0.0679 (17)	0.0558 (16)	0.0735 (19)	0.0105 (13)	0.0309 (15)	0.0275 (14)
C54	0.0661 (17)	0.0415 (13)	0.079 (2)	0.0066 (12)	0.0284 (15)	0.0074 (12)
C55	0.0788 (19)	0.0509 (14)	0.0501 (14)	0.0075 (13)	0.0199 (13)	0.0026 (12)
C56	0.0750 (18)	0.0475 (14)	0.0550 (15)	0.0093 (12)	0.0313 (13)	0.0121 (11)
O54	0.0909 (17)	0.0512 (12)	0.120 (2)	−0.0097 (11)	0.0182 (15)	0.0105 (12)
C57	0.094 (3)	0.077 (2)	0.115 (3)	−0.020 (2)	0.013 (2)	−0.011 (2)

### (RS)-5-(4-Methoxyphenyl)-1-(4-phenythiazol-2-yl)-3-(4-(prop-2-ynyloxy)phenyl)-4,5-dihydro-1H-pyrazole (II) Geometric parameters (Å, º)

**Table:**

N1—C12	1.363 (4)	C32—C33	1.365 (4)
N1—N2	1.383 (3)	C32—H32	0.9300
N1—C5	1.468 (4)	C33—C34	1.401 (4)
N2—C3	1.289 (4)	C33—H33	0.9300
C3—C31	1.455 (4)	C34—O34	1.372 (3)
C3—C4	1.505 (4)	C34—C35	1.373 (4)
C4—C5	1.543 (4)	C35—C36	1.378 (4)
C4—H4A	0.9700	C35—H35	0.9300
C4—H4B	0.9700	C36—H36	0.9300
C5—C51	1.513 (4)	O34—C37	1.430 (4)
C5—H5	0.9800	C37—C38	1.443 (5)
S11—C15	1.729 (4)	C37—H37A	0.9700
S11—C12	1.747 (3)	C37—H37B	0.9700
C12—N13	1.298 (4)	C38—C39	1.162 (5)
N13—C14	1.395 (3)	C39—H39	0.9300
C14—C15	1.361 (5)	C51—C56	1.375 (4)
C14—C141	1.471 (5)	C51—C52	1.393 (4)
C15—H15	0.9300	C52—C53	1.367 (4)
C141—C142	1.388 (5)	C52—H52	0.9300
C141—C146	1.397 (4)	C53—C54	1.385 (4)
C142—C143	1.379 (5)	C53—H53	0.9300
C142—H142	0.9300	C54—O54	1.371 (4)
C143—C144	1.370 (5)	C54—C55	1.374 (4)
C143—H143	0.9300	C55—C56	1.384 (4)
C144—C145	1.365 (6)	C55—H155	0.9300
C144—H144	0.9300	C56—H56	0.9300
C145—C146	1.383 (6)	O54—C57	1.412 (5)
C145—H145	0.9300	C57—H57A	0.9600
C146—H146	0.9300	C57—H57B	0.9600
C31—C36	1.396 (4)	C57—H57C	0.9600
C31—C32	1.400 (4)		
			
C12—N1—N2	119.8 (2)	C32—C31—C3	122.1 (2)
C12—N1—C5	125.0 (3)	C33—C32—C31	121.4 (3)
N2—N1—C5	114.1 (2)	C33—C32—H32	119.3
C3—N2—N1	108.0 (2)	C31—C32—H32	119.3
N2—C3—C31	122.7 (2)	C32—C33—C34	119.7 (3)
N2—C3—C4	113.6 (2)	C32—C33—H33	120.1
C31—C3—C4	123.7 (2)	C34—C33—H33	120.1
C3—C4—C5	102.9 (2)	O34—C34—C35	124.5 (3)
C3—C4—H4A	111.2	O34—C34—C33	115.6 (2)
C5—C4—H4A	111.2	C35—C34—C33	119.9 (3)
C3—C4—H4B	111.2	C34—C35—C36	119.9 (2)
C5—C4—H4B	111.2	C34—C35—H35	120.1
H4A—C4—H4B	109.1	C36—C35—H35	120.1
N1—C5—C51	114.1 (2)	C35—C36—C31	121.4 (3)
N1—C5—C4	100.8 (2)	C35—C36—H36	119.3
C51—C5—C4	111.9 (2)	C31—C36—H36	119.3
N1—C5—H5	109.9	C34—O34—C37	116.3 (2)
C51—C5—H5	109.9	O34—C37—C38	109.3 (3)
C4—C5—H5	109.9	O34—C37—H37A	109.8
C15—S11—C12	87.71 (16)	C38—C37—H37A	109.8
N13—C12—N1	123.6 (3)	O34—C37—H37B	109.8
N13—C12—S11	116.2 (2)	C38—C37—H37B	109.8
N1—C12—S11	120.2 (3)	H37A—C37—H37B	108.3
C12—N13—C14	110.1 (3)	C39—C38—C37	175.7 (4)
C15—C14—N13	114.7 (3)	C38—C39—H39	180.0
C15—C14—C141	126.4 (3)	C56—C51—C52	117.8 (3)
N13—C14—C141	118.9 (3)	C56—C51—C5	124.3 (2)
C14—C15—S11	111.4 (2)	C52—C51—C5	117.8 (2)
C14—C15—H15	124.3	C53—C52—C51	121.3 (3)
S11—C15—H15	124.3	C53—C52—H52	119.3
C142—C141—C146	117.0 (4)	C51—C52—H52	119.3
C142—C141—C14	121.0 (3)	C52—C53—C54	120.1 (2)
C146—C141—C14	121.9 (3)	C52—C53—H53	119.9
C143—C142—C141	121.1 (3)	C54—C53—H53	119.9
C143—C142—H142	119.4	O54—C54—C55	124.5 (3)
C141—C142—H142	119.4	O54—C54—C53	116.2 (3)
C144—C143—C142	121.0 (4)	C55—C54—C53	119.3 (3)
C144—C143—H143	119.5	C54—C55—C56	120.1 (3)
C142—C143—H143	119.5	C54—C55—H155	119.9
C145—C144—C143	119.1 (4)	C56—C55—H155	119.9
C145—C144—H144	120.5	C51—C56—C55	121.2 (2)
C143—C144—H144	120.5	C51—C56—H56	119.4
C144—C145—C146	120.7 (3)	C55—C56—H56	119.4
C144—C145—H145	119.7	C54—O54—C57	117.5 (3)
C146—C145—H145	119.7	O54—C57—H57A	109.5
C145—C146—C141	121.1 (4)	O54—C57—H57B	109.5
C145—C146—H146	119.5	H57A—C57—H57B	109.5
C141—C146—H146	119.5	O54—C57—H57C	109.5
C36—C31—C32	117.6 (3)	H57A—C57—H57C	109.5
C36—C31—C3	120.3 (2)	H57B—C57—H57C	109.5
			
C12—N1—N2—C3	166.6 (3)	C142—C141—C146—C145	−0.4 (5)
C5—N1—N2—C3	−2.0 (3)	C14—C141—C146—C145	178.5 (3)
N1—N2—C3—C31	178.9 (2)	N2—C3—C31—C36	−172.2 (2)
N1—N2—C3—C4	−3.5 (3)	C4—C3—C31—C36	10.5 (4)
N2—C3—C4—C5	7.2 (3)	N2—C3—C31—C32	9.5 (4)
C31—C3—C4—C5	−175.2 (2)	C4—C3—C31—C32	−167.9 (3)
C12—N1—C5—C51	78.2 (4)	C36—C31—C32—C33	−0.4 (4)
N2—N1—C5—C51	−113.9 (3)	C3—C31—C32—C33	178.0 (3)
C12—N1—C5—C4	−161.8 (3)	C31—C32—C33—C34	0.8 (4)
N2—N1—C5—C4	6.1 (3)	C32—C33—C34—O34	−179.4 (3)
C3—C4—C5—N1	−7.3 (3)	C32—C33—C34—C35	−0.1 (4)
C3—C4—C5—C51	114.4 (2)	O34—C34—C35—C36	178.2 (3)
N2—N1—C12—N13	−178.5 (3)	C33—C34—C35—C36	−1.0 (4)
C5—N1—C12—N13	−11.2 (5)	C34—C35—C36—C31	1.5 (4)
N2—N1—C12—S11	2.5 (4)	C32—C31—C36—C35	−0.7 (4)
C5—N1—C12—S11	169.8 (2)	C3—C31—C36—C35	−179.1 (2)
C15—S11—C12—N13	−1.3 (2)	C35—C34—O34—C37	−0.6 (4)
C15—S11—C12—N1	177.9 (3)	C33—C34—O34—C37	178.6 (3)
N1—C12—N13—C14	−178.1 (3)	C34—O34—C37—C38	−174.0 (3)
S11—C12—N13—C14	1.0 (3)	N1—C5—C51—C56	16.6 (4)
C12—N13—C14—C15	−0.2 (4)	C4—C5—C51—C56	−97.0 (3)
C12—N13—C14—C141	178.7 (2)	N1—C5—C51—C52	−167.0 (2)
N13—C14—C15—S11	−0.8 (3)	C4—C5—C51—C52	79.4 (3)
C141—C14—C15—S11	−179.5 (2)	C56—C51—C52—C53	−1.4 (4)
C12—S11—C15—C14	1.1 (2)	C5—C51—C52—C53	−178.0 (3)
C15—C14—C141—C142	−174.7 (3)	C51—C52—C53—C54	−1.2 (4)
N13—C14—C141—C142	6.6 (4)	C52—C53—C54—O54	−176.3 (3)
C15—C14—C141—C146	6.3 (5)	C52—C53—C54—C55	2.7 (4)
N13—C14—C141—C146	−172.4 (3)	O54—C54—C55—C56	177.2 (3)
C146—C141—C142—C143	1.1 (5)	C53—C54—C55—C56	−1.7 (4)
C14—C141—C142—C143	−177.9 (3)	C52—C51—C56—C55	2.5 (4)
C141—C142—C143—C144	−0.8 (5)	C5—C51—C56—C55	178.9 (3)
C142—C143—C144—C145	−0.1 (6)	C54—C55—C56—C51	−1.0 (4)
C143—C144—C145—C146	0.8 (6)	C55—C54—O54—C57	−21.0 (5)
C144—C145—C146—C141	−0.5 (5)	C53—C54—O54—C57	157.9 (3)

### (RS)-5-(4-Methoxyphenyl)-1-(4-phenythiazol-2-yl)-3-(4-(prop-2-ynyloxy)phenyl)-4,5-dihydro-1H-pyrazole (II) Hydrogen-bond geometry (Å, º)

**Table:**

*D*—H···*A*	*D*—H	H···*A*	*D*···*A*	*D*—H···*A*
C56—H56···N1	0.93	2.59	2.915 (4)	101
C142—H142···N13	0.93	2.53	2.864 (5)	101
C39—H39···*Cg*2^i^	0.93	2.59	3.365 (5)	141
C56—H56···*Cg*1^ii^	0.93	2.91	3.688 (3)	142

Symmetry codes: (i) *x*+1, *y*, *z*; (ii) *x*−1/2, −*y*+1/2, *z*−1/2.

### (RS)-5-(4-Methoxyphenyl)-1-[4-(4-methylphenyl)thiazol-2-yl]-3-[4-(prop-2-ynyloxy)phenyl]-4,5-dihydro-1H-pyrazole (III) Crystal data

**Table:**

C_29_H_25_N_3_O_2_S	*F*(000) = 1008
*M**_r_* = 479.58	*D*_x_ = 1.288 Mg m^−^^3^
Monoclinic, *C**c*	Mo *K*α radiation, λ = 0.71073 Å
*a* = 16.5634 (17) Å	Cell parameters from 5580 reflections
*b* = 17.7250 (19) Å	θ = 2.5–27.6°
*c* = 9.4032 (11) Å	µ = 0.16 mm^−^^1^
β = 116.401 (3)°	*T* = 297 K
*V* = 2472.7 (5) Å^3^	Block, colourless
*Z* = 4	0.18 × 0.16 × 0.15 mm

### (RS)-5-(4-Methoxyphenyl)-1-[4-(4-methylphenyl)thiazol-2-yl]-3-[4-(prop-2-ynyloxy)phenyl]-4,5-dihydro-1H-pyrazole (III) Data collection

**Table:**

Bruker D8 Venture diffractometer	5578 independent reflections
Radiation source: INCOATEC high brilliance microfocus sealed tube	3802 reflections with *I* > 2σ(*I*)
Multilayer mirror monochromator	*R*_int_ = 0.058
φ and ω scans	θ_max_ = 27.6°, θ_min_ = 3.3°
Absorption correction: multi-scan (SADABS; Bruker, 2016)	*h* = −21→20
*T*_min_ = 0.949, *T*_max_ = 0.976	*k* = −22→23
40416 measured reflections	*l* = −12→12

### (RS)-5-(4-Methoxyphenyl)-1-[4-(4-methylphenyl)thiazol-2-yl]-3-[4-(prop-2-ynyloxy)phenyl]-4,5-dihydro-1H-pyrazole (III) Refinement

**Table:**

Refinement on *F*^2^	Hydrogen site location: inferred from neighbouring sites
Least-squares matrix: full	H-atom parameters constrained
*R*[*F*^2^ > 2σ(*F*^2^)] = 0.042	*w* = 1/[σ^2^(*F*_o_^2^) + (0.0501*P*)^2^ + 0.6209*P*] where *P* = (*F*_o_^2^ + 2*F*_c_^2^)/3
*wR*(*F*^2^) = 0.121	(Δ/σ)_max_ < 0.001
*S* = 1.08	Δρ_max_ = 0.15 e Å^−^^3^
5578 reflections	Δρ_min_ = −0.17 e Å^−^^3^
318 parameters	Absolute structure: Flack *x* determined using 1613 quotients [(*I*^+^)-(*I*^-^)]/[(*I*^+^)+(*I*^-^)] (Parsons *et al.*, 2013)
2 restraints	Absolute structure parameter: −0.01 (3)
Primary atom site location: difference Fourier map	

### (RS)-5-(4-Methoxyphenyl)-1-[4-(4-methylphenyl)thiazol-2-yl]-3-[4-(prop-2-ynyloxy)phenyl]-4,5-dihydro-1H-pyrazole (III) Special details

**Table:**

Geometry. All esds (except the esd in the dihedral angle between two l.s. planes) are estimated using the full covariance matrix. The cell esds are taken into account individually in the estimation of esds in distances, angles and torsion angles; correlations between esds in cell parameters are only used when they are defined by crystal symmetry. An approximate (isotropic) treatment of cell esds is used for estimating esds involving l.s. planes.

### (RS)-5-(4-Methoxyphenyl)-1-[4-(4-methylphenyl)thiazol-2-yl]-3-[4-(prop-2-ynyloxy)phenyl]-4,5-dihydro-1H-pyrazole (III) Fractional atomic coordinates and isotropic or equivalent isotropic displacement parameters (Å^2^)

**Table:**

	*x*	*y*	*z*	*U*_iso_*/*U*_eq_	
N1	0.3744 (3)	0.29841 (18)	0.5726 (4)	0.0839 (9)	
N2	0.4454 (2)	0.30917 (17)	0.5355 (4)	0.0737 (8)	
C3	0.5038 (3)	0.25572 (19)	0.6007 (4)	0.0669 (9)	
C4	0.4776 (3)	0.2029 (2)	0.6985 (5)	0.0865 (12)	
H4A	0.5215	0.2044	0.8093	0.104*	
H4B	0.4725	0.1515	0.6600	0.104*	
C5	0.3853 (3)	0.2328 (2)	0.6773 (5)	0.0756 (10)	
H5	0.3904	0.2505	0.7797	0.091*	
S11	0.32183 (9)	0.44056 (5)	0.46246 (13)	0.0854 (3)	
C12	0.3157 (3)	0.35575 (19)	0.5522 (5)	0.0724 (10)	
N13	0.2519 (2)	0.35085 (16)	0.5945 (4)	0.0747 (9)	
C14	0.1990 (3)	0.41597 (19)	0.5524 (4)	0.0709 (11)	
C15	0.2266 (3)	0.4692 (2)	0.4798 (5)	0.0824 (12)	
H15	0.1979	0.5153	0.4437	0.099*	
C141	0.1221 (3)	0.4193 (2)	0.5878 (4)	0.0713 (10)	
C142	0.1041 (3)	0.3605 (2)	0.6669 (5)	0.0872 (12)	
H142	0.1428	0.3192	0.6997	0.105*	
C143	0.0302 (4)	0.3619 (3)	0.6979 (6)	0.0973 (14)	
H143	0.0210	0.3216	0.7523	0.117*	
C144	−0.0303 (3)	0.4203 (3)	0.6517 (5)	0.0905 (13)	
C145	−0.0122 (4)	0.4792 (3)	0.5748 (6)	0.0996 (16)	
H145	−0.0509	0.5205	0.5439	0.120*	
C146	0.0608 (4)	0.4798 (2)	0.5416 (6)	0.0942 (14)	
H146	0.0697	0.5206	0.4881	0.113*	
C147	−0.1117 (4)	0.4210 (4)	0.6812 (8)	0.126 (2)	
H14A	−0.0998	0.4509	0.7735	0.190*	
H14B	−0.1260	0.3703	0.6981	0.190*	
H14C	−0.1616	0.4422	0.5909	0.190*	
C31	0.5854 (2)	0.2478 (2)	0.5802 (4)	0.0656 (9)	
C32	0.6106 (3)	0.3016 (2)	0.4991 (4)	0.0738 (10)	
H32	0.5744	0.3438	0.4569	0.089*	
C33	0.6869 (3)	0.2937 (2)	0.4803 (5)	0.0773 (11)	
H33	0.7023	0.3303	0.4258	0.093*	
C34	0.7425 (3)	0.2308 (2)	0.5428 (4)	0.0717 (10)	
C35	0.7184 (3)	0.1771 (2)	0.6216 (5)	0.0769 (11)	
H35	0.7544	0.1347	0.6621	0.092*	
C36	0.6411 (3)	0.1854 (2)	0.6415 (5)	0.0751 (10)	
H36	0.6261	0.1488	0.6966	0.090*	
O34	0.8184 (2)	0.22817 (16)	0.5206 (3)	0.0846 (8)	
C37	0.8746 (3)	0.1632 (3)	0.5821 (7)	0.1023 (15)	
H37A	0.8416	0.1183	0.5288	0.123*	
H37B	0.8916	0.1582	0.6946	0.123*	
C38	0.9547 (4)	0.1703 (3)	0.5582 (6)	0.0952 (14)	
C39	1.0224 (5)	0.1735 (3)	0.5477 (7)	0.1132 (18)	
H39	1.0762	0.1761	0.5393	0.136*	
C51	0.3113 (2)	0.17528 (19)	0.6090 (4)	0.0651 (9)	
C52	0.2983 (3)	0.1276 (2)	0.7136 (5)	0.0749 (10)	
H52	0.3335	0.1335	0.8222	0.090*	
C53	0.2344 (3)	0.0719 (3)	0.6598 (5)	0.0817 (11)	
H53	0.2273	0.0402	0.7322	0.098*	
C54	0.1803 (3)	0.0622 (2)	0.4989 (5)	0.0739 (10)	
C55	0.1924 (3)	0.1084 (2)	0.3941 (5)	0.0832 (12)	
H155	0.1568	0.1025	0.2856	0.100*	
C56	0.2579 (3)	0.1643 (2)	0.4491 (5)	0.0789 (11)	
H56	0.2660	0.1950	0.3762	0.095*	
O54	0.1169 (2)	0.00585 (18)	0.4582 (4)	0.1059 (11)	
C57	0.0440 (4)	0.0079 (3)	0.3046 (7)	0.1147 (17)	
H57A	0.0655	−0.0025	0.2272	0.172*	
H57B	0.0167	0.0570	0.2854	0.172*	
H57C	0.0002	−0.0294	0.2969	0.172*	

### (RS)-5-(4-Methoxyphenyl)-1-[4-(4-methylphenyl)thiazol-2-yl]-3-[4-(prop-2-ynyloxy)phenyl]-4,5-dihydro-1H-pyrazole (III) Atomic displacement parameters (Å^2^)

**Table:**

	*U*^11^	*U*^22^	*U*^33^	*U*^12^	*U*^13^	*U*^23^
N1	0.099 (2)	0.0566 (18)	0.098 (3)	0.0104 (16)	0.046 (2)	0.0159 (16)
N2	0.091 (2)	0.0502 (15)	0.073 (2)	0.0009 (15)	0.0296 (17)	0.0010 (14)
C3	0.082 (2)	0.0499 (17)	0.0531 (19)	−0.0041 (16)	0.0163 (16)	−0.0014 (14)
C4	0.089 (3)	0.069 (2)	0.094 (3)	0.009 (2)	0.033 (2)	0.023 (2)
C5	0.091 (3)	0.060 (2)	0.073 (2)	0.0107 (19)	0.034 (2)	0.0101 (17)
S11	0.1088 (8)	0.0476 (4)	0.0845 (6)	−0.0078 (5)	0.0290 (5)	−0.0009 (5)
C12	0.087 (3)	0.0471 (18)	0.069 (2)	0.0006 (17)	0.022 (2)	0.0008 (16)
N13	0.092 (2)	0.0472 (15)	0.071 (2)	0.0099 (15)	0.0238 (17)	0.0051 (13)
C14	0.089 (3)	0.0447 (17)	0.056 (2)	0.0020 (16)	0.0108 (18)	−0.0061 (15)
C15	0.102 (3)	0.0448 (17)	0.083 (3)	0.0007 (19)	0.025 (2)	−0.0027 (18)
C141	0.086 (3)	0.0486 (17)	0.057 (2)	0.0062 (17)	0.0117 (18)	−0.0087 (15)
C142	0.108 (3)	0.068 (2)	0.079 (3)	0.025 (2)	0.036 (2)	0.011 (2)
C143	0.121 (4)	0.081 (3)	0.086 (3)	0.013 (3)	0.042 (3)	0.003 (2)
C144	0.093 (3)	0.081 (3)	0.078 (3)	0.007 (2)	0.021 (2)	−0.023 (2)
C145	0.092 (3)	0.079 (3)	0.102 (4)	0.025 (3)	0.019 (3)	−0.013 (3)
C146	0.108 (4)	0.057 (2)	0.091 (3)	0.013 (2)	0.020 (3)	0.005 (2)
C147	0.107 (4)	0.138 (5)	0.123 (5)	0.005 (4)	0.041 (3)	−0.042 (4)
C31	0.075 (2)	0.0540 (18)	0.0511 (19)	−0.0016 (16)	0.0130 (16)	−0.0023 (14)
C32	0.081 (3)	0.0548 (19)	0.069 (2)	0.0055 (17)	0.0191 (19)	0.0100 (16)
C33	0.084 (3)	0.064 (2)	0.074 (3)	0.0034 (19)	0.025 (2)	0.0148 (19)
C34	0.083 (3)	0.057 (2)	0.061 (2)	0.0065 (17)	0.0198 (19)	0.0017 (16)
C35	0.088 (3)	0.055 (2)	0.069 (2)	0.0114 (18)	0.019 (2)	0.0077 (16)
C36	0.093 (3)	0.0531 (19)	0.068 (2)	0.0006 (18)	0.025 (2)	0.0067 (16)
O34	0.0885 (19)	0.0718 (17)	0.0859 (18)	0.0176 (15)	0.0320 (15)	0.0155 (13)
C37	0.107 (4)	0.071 (3)	0.121 (4)	0.021 (2)	0.044 (3)	0.018 (3)
C38	0.105 (4)	0.079 (3)	0.090 (3)	0.033 (3)	0.033 (3)	0.010 (2)
C39	0.104 (4)	0.118 (4)	0.110 (4)	0.045 (3)	0.041 (3)	0.015 (3)
C51	0.078 (2)	0.0539 (17)	0.063 (2)	0.0140 (15)	0.0306 (17)	0.0074 (15)
C52	0.076 (2)	0.087 (3)	0.058 (2)	0.009 (2)	0.0264 (18)	0.0138 (18)
C53	0.080 (3)	0.088 (3)	0.076 (3)	0.011 (2)	0.034 (2)	0.028 (2)
C54	0.073 (2)	0.0547 (19)	0.086 (3)	0.0147 (18)	0.028 (2)	0.0107 (18)
C55	0.106 (3)	0.065 (2)	0.063 (2)	0.004 (2)	0.023 (2)	0.0024 (19)
C56	0.114 (3)	0.061 (2)	0.061 (2)	0.003 (2)	0.039 (2)	0.0118 (17)
O54	0.093 (2)	0.078 (2)	0.116 (3)	−0.0087 (16)	0.0193 (19)	0.0156 (17)
C57	0.101 (4)	0.090 (3)	0.120 (4)	−0.012 (3)	0.020 (3)	−0.010 (3)

### (RS)-5-(4-Methoxyphenyl)-1-[4-(4-methylphenyl)thiazol-2-yl]-3-[4-(prop-2-ynyloxy)phenyl]-4,5-dihydro-1H-pyrazole (III) Geometric parameters (Å, º)

**Table:**

N1—C12	1.359 (5)	C31—C36	1.391 (5)
N1—N2	1.381 (5)	C31—C32	1.396 (5)
N1—C5	1.482 (5)	C32—C33	1.359 (6)
N2—C3	1.297 (5)	C32—H32	0.9300
C3—C31	1.454 (5)	C33—C34	1.399 (5)
C3—C4	1.505 (6)	C33—H33	0.9300
C4—C5	1.544 (6)	C34—O34	1.364 (5)
C4—H4A	0.9700	C34—C35	1.369 (6)
C4—H4B	0.9700	C35—C36	1.382 (6)
C5—C51	1.501 (5)	C35—H35	0.9300
C5—H5	0.9800	C36—H36	0.9300
S11—C15	1.732 (5)	O34—C37	1.431 (5)
S11—C12	1.749 (4)	C37—C38	1.447 (7)
C12—N13	1.287 (5)	C37—H37A	0.9700
N13—C14	1.396 (5)	C37—H37B	0.9700
C14—C15	1.357 (6)	C38—C39	1.169 (8)
C14—C141	1.454 (6)	C39—H39	0.9300
C15—H15	0.9300	C51—C56	1.378 (5)
C141—C142	1.388 (6)	C51—C52	1.384 (5)
C141—C146	1.406 (6)	C52—C53	1.369 (6)
C142—C143	1.379 (6)	C52—H52	0.9300
C142—H142	0.9300	C53—C54	1.384 (6)
C143—C144	1.371 (7)	C53—H53	0.9300
C143—H143	0.9300	C54—C55	1.364 (6)
C144—C145	1.377 (8)	C54—O54	1.374 (5)
C144—C147	1.492 (8)	C55—C56	1.387 (6)
C145—C146	1.376 (7)	C55—H155	0.9300
C145—H145	0.9300	C56—H56	0.9300
C146—H146	0.9300	O54—C57	1.412 (7)
C147—H14A	0.9600	C57—H57A	0.9600
C147—H14B	0.9600	C57—H57B	0.9600
C147—H14C	0.9600	C57—H57C	0.9600
			
C12—N1—N2	119.8 (3)	H14B—C147—H14C	109.5
C12—N1—C5	123.3 (3)	C36—C31—C32	117.7 (4)
N2—N1—C5	114.2 (3)	C36—C31—C3	120.4 (3)
C3—N2—N1	108.6 (3)	C32—C31—C3	121.9 (3)
N2—C3—C31	123.3 (3)	C33—C32—C31	121.5 (3)
N2—C3—C4	112.7 (4)	C33—C32—H32	119.3
C31—C3—C4	124.0 (3)	C31—C32—H32	119.3
C3—C4—C5	104.2 (3)	C32—C33—C34	120.2 (4)
C3—C4—H4A	110.9	C32—C33—H33	119.9
C5—C4—H4A	110.9	C34—C33—H33	119.9
C3—C4—H4B	110.9	O34—C34—C35	124.7 (3)
C5—C4—H4B	110.9	O34—C34—C33	116.2 (4)
H4A—C4—H4B	108.9	C35—C34—C33	119.1 (4)
N1—C5—C51	114.4 (3)	C34—C35—C36	120.6 (3)
N1—C5—C4	100.2 (3)	C34—C35—H35	119.7
C51—C5—C4	113.3 (3)	C36—C35—H35	119.7
N1—C5—H5	109.5	C35—C36—C31	120.8 (4)
C51—C5—H5	109.5	C35—C36—H36	119.6
C4—C5—H5	109.5	C31—C36—H36	119.6
C15—S11—C12	87.5 (2)	C34—O34—C37	116.3 (3)
N13—C12—N1	122.6 (3)	O34—C37—C38	110.1 (4)
N13—C12—S11	116.0 (3)	O34—C37—H37A	109.6
N1—C12—S11	121.3 (3)	C38—C37—H37A	109.6
C12—N13—C14	110.8 (3)	O34—C37—H37B	109.6
C15—C14—N13	114.0 (4)	C38—C37—H37B	109.6
C15—C14—C141	127.8 (4)	H37A—C37—H37B	108.2
N13—C14—C141	118.2 (3)	C39—C38—C37	175.7 (5)
C14—C15—S11	111.6 (3)	C38—C39—H39	180.0
C14—C15—H15	124.2	C56—C51—C52	117.5 (4)
S11—C15—H15	124.2	C56—C51—C5	124.6 (3)
C142—C141—C146	116.1 (4)	C52—C51—C5	117.8 (3)
C142—C141—C14	120.7 (3)	C53—C52—C51	121.1 (4)
C146—C141—C14	123.1 (4)	C53—C52—H52	119.4
C143—C142—C141	121.5 (4)	C51—C52—H52	119.4
C143—C142—H142	119.3	C52—C53—C54	120.8 (4)
C141—C142—H142	119.3	C52—C53—H53	119.6
C144—C143—C142	122.6 (5)	C54—C53—H53	119.6
C144—C143—H143	118.7	C55—C54—O54	125.1 (4)
C142—C143—H143	118.7	C55—C54—C53	118.9 (4)
C143—C144—C145	116.1 (5)	O54—C54—C53	116.0 (4)
C143—C144—C147	122.7 (6)	C54—C55—C56	120.1 (4)
C145—C144—C147	121.2 (5)	C54—C55—H155	119.9
C146—C145—C144	122.9 (4)	C56—C55—H155	119.9
C146—C145—H145	118.5	C51—C56—C55	121.6 (4)
C144—C145—H145	118.5	C51—C56—H56	119.2
C145—C146—C141	120.7 (5)	C55—C56—H56	119.2
C145—C146—H146	119.6	C54—O54—C57	117.8 (4)
C141—C146—H146	119.6	O54—C57—H57A	109.5
C144—C147—H14A	109.5	O54—C57—H57B	109.5
C144—C147—H14B	109.5	H57A—C57—H57B	109.5
H14A—C147—H14B	109.5	O54—C57—H57C	109.5
C144—C147—H14C	109.5	H57A—C57—H57C	109.5
H14A—C147—H14C	109.5	H57B—C57—H57C	109.5
			
C12—N1—N2—C3	163.9 (3)	C144—C145—C146—C141	−0.9 (7)
C5—N1—N2—C3	1.9 (4)	C142—C141—C146—C145	0.1 (6)
N1—N2—C3—C31	177.2 (3)	C14—C141—C146—C145	178.4 (4)
N1—N2—C3—C4	−2.4 (5)	N2—C3—C31—C36	−174.1 (3)
N2—C3—C4—C5	2.0 (5)	C4—C3—C31—C36	5.5 (5)
C31—C3—C4—C5	−177.7 (3)	N2—C3—C31—C32	5.7 (5)
C12—N1—C5—C51	76.7 (5)	C4—C3—C31—C32	−174.7 (4)
N2—N1—C5—C51	−122.1 (4)	C36—C31—C32—C33	−0.1 (5)
C12—N1—C5—C4	−161.9 (4)	C3—C31—C32—C33	−179.9 (3)
N2—N1—C5—C4	−0.6 (4)	C31—C32—C33—C34	0.0 (6)
C3—C4—C5—N1	−0.7 (4)	C32—C33—C34—O34	−179.0 (3)
C3—C4—C5—C51	121.6 (3)	C32—C33—C34—C35	0.6 (6)
N2—N1—C12—N13	−175.2 (4)	O34—C34—C35—C36	178.5 (4)
C5—N1—C12—N13	−15.0 (6)	C33—C34—C35—C36	−1.0 (6)
N2—N1—C12—S11	6.0 (5)	C34—C35—C36—C31	0.9 (6)
C5—N1—C12—S11	166.3 (3)	C32—C31—C36—C35	−0.4 (5)
C15—S11—C12—N13	−2.1 (3)	C3—C31—C36—C35	179.5 (3)
C15—S11—C12—N1	176.8 (3)	C35—C34—O34—C37	1.5 (6)
N1—C12—N13—C14	−176.8 (4)	C33—C34—O34—C37	−179.0 (4)
S11—C12—N13—C14	2.0 (4)	C34—O34—C37—C38	−176.6 (4)
C12—N13—C14—C15	−0.8 (4)	N1—C5—C51—C56	25.0 (5)
C12—N13—C14—C141	178.5 (3)	C4—C5—C51—C56	−89.0 (4)
N13—C14—C15—S11	−0.7 (4)	N1—C5—C51—C52	−158.0 (3)
C141—C14—C15—S11	−179.9 (3)	C4—C5—C51—C52	88.0 (4)
C12—S11—C15—C14	1.4 (3)	C56—C51—C52—C53	−0.3 (6)
C15—C14—C141—C142	−178.4 (4)	C5—C51—C52—C53	−177.5 (4)
N13—C14—C141—C142	2.4 (5)	C51—C52—C53—C54	−0.7 (6)
C15—C14—C141—C146	3.5 (6)	C52—C53—C54—C55	1.0 (6)
N13—C14—C141—C146	−175.7 (4)	C52—C53—C54—O54	−178.4 (4)
C146—C141—C142—C143	−0.1 (6)	O54—C54—C55—C56	179.1 (4)
C14—C141—C142—C143	−178.3 (4)	C53—C54—C55—C56	−0.3 (6)
C141—C142—C143—C144	0.8 (7)	C52—C51—C56—C55	1.0 (6)
C142—C143—C144—C145	−1.5 (7)	C5—C51—C56—C55	178.0 (4)
C142—C143—C144—C147	178.3 (5)	C54—C55—C56—C51	−0.7 (7)
C143—C144—C145—C146	1.5 (7)	C55—C54—O54—C57	−17.9 (7)
C147—C144—C145—C146	−178.2 (4)	C53—C54—O54—C57	161.5 (4)

### (RS)-5-(4-Methoxyphenyl)-1-[4-(4-methylphenyl)thiazol-2-yl]-3-[4-(prop-2-ynyloxy)phenyl]-4,5-dihydro-1H-pyrazole (III) Hydrogen-bond geometry (Å, º)

**Table:**

*D*—H···*A*	*D*—H	H···*A*	*D*···*A*	*D*—H···*A*
C142—H142···N13	0.93	2.48	2.823 (6)	102
C35—H35···S11^i^	0.93	2.86	3.560 (4)	133
C39—H39···*Cg*2^ii^	0.93	2.93	3.802 (5)	156
C56—H56···*Cg*1^iii^	0.93	2.92	3.689 (3)	141

Symmetry codes: (i) *x*+1/2, −*y*+1/2, *z*+1/2; (ii) *x*+1, *y*, *z*; (iii) *x*−1/2, −*y*+1/2, *z*−1/2.
